# Spinal block and delirium in oncologic patients after laparoscopic surgery in the Trendelenburg position: A randomized controlled trial

**DOI:** 10.1371/journal.pone.0249808

**Published:** 2021-05-17

**Authors:** Jorge Kiyoshi Mitsunaga, Vinicius Fernando Calsavara, Elton Shinji Onari, Vinicius Monteiro Arantes, Carolina Paiva Akamine, Adriana Mayumi Handa, Michael Madeira de la Cruz Quezada, Franco Yasuhiro Ito, Ana Carolina Souza Porto, Eduardo Henrique Giroud Joaquim, Giane Nakamura

**Affiliations:** 1 Department of Anaesthesiology, A.C.Camargo Cancer Center, São Paulo, Brazil; 2 Department of Epidemiology and Statistics, A.C.Camargo Cancer Center, São Paulo, Brazil; 3 Department of Cutaneous Oncology, A.C.Camargo Cancer Center, São Paulo, Brazil; Public Library of Science, UNITED KINGDOM

## Abstract

Delirium is the most common postsurgical neurological complication and has a variable incidence rate. Laparoscopic surgery, when associated with the Trendelenburg position, can cause innumerable physiological changes and increase the risk of neurocognitive changes. The association of general anesthesia with a spinal block allows the use of lower doses of anesthetic agents for anesthesia maintenance and facilitates better control over postoperative pain. Our primary outcome was to assess whether a spinal block influences the incidence of delirium in oncologic patients following laparoscopic surgery in the Trendelenburg position. Our secondary outcome was to analyze whether there were other associated factors. A total of 150 oncologic patients who underwent elective laparoscopic surgeries in the Trendelenburg position were included in this randomized controlled trial. The patients were randomized into 2 groups: the general anesthesia group and the general anesthesia plus spinal block group. Patients were immediately evaluated during the postoperative period and monitored until they were discharged, to rule out the presence of delirium. Delirium occurred in 29 patients in total (22.3%) (general anesthesia group: 30.8%; general anesthesia plus spinal block: 13.8% p = 0.035). Patients who received general anesthesia had a higher risk of delirium than patients who received general anesthesia associated with a spinal block (odds ratio = 3.4; 95% confidence interval: 1.2–9.6; p = 0.020). Spinal block was associated with reduced delirium incidence in oncologic patients who underwent elective laparoscopic surgeries in the Trendelenburg position.

## Introduction

The nomenclature associated with cognitive disorders that develop during the perioperative period has always been controversial. In 2018, based on the Diagnostic and Statistical Manual of Mental Disorders, fifth edition (DSM-5), “perioperative neurocognitive disorders” was established as the standard term encompassing all cognitive disorders, both preoperative and postoperative [1].

Emergence delirium is a neurocognitive disorder (NCD) that develops after extubation without any period of lucidity having occurred. “Postoperative delirium” (PD) is a condition that occurs up to 1 week post-procedure or until discharge (whichever occurs first) [1]. PD is considered the most common postoperative neurological complication [2]. It has significantly variable incidence among studies [3–5], and several risk factors have been reported in the literature [6, 7]. Some of these risk factors have greater supporting evidence, such as the presence of pre-existing cognitive impairment [6, 8], advanced age [9, 10], and emergency [6], cardiac [11], and orthopedic surgeries [6].

The Trendelenburg position (TP) is a supine position, with the head of the operating table tilted down. Trendelenburg positioning improves the exposure of pelvic organs during laparoscopic surgery. When associated with the TP, laparoscopic surgery entails several physiological changes [12–15] and could be a risk factor for the development of delirium.

The combination of general anesthesia with a spinal block (SB) allows for lower doses of anesthetic agents to maintain anesthesia, and enables better control over postoperative pain [16–18], early awakenings, decreased nausea and vomiting, decreased length of hospital stay, and improved patient satisfaction [19–22].

This study evaluated whether cancer patients undergoing laparoscopic surgery in the TP under general anesthesia exhibited a different incidence of delirium compared to patients undergoing the same procedure, but associated with a SB.

## Methods

A prospective, randomized study was conducted between October, 2017, and October, 2018, per the Declaration of Helsinki. It was approved by the A.C.Camargo Cancer Center Institucional Ethics Committee (number: 2205/16) and registered in the Brazilian Clinical Trials Registry (ReBec; RBR-9yrqwh). Written informed consent was obtained from patients before inclusion. The CONSORT guidelines were used for article preparation.

### Study population

**Fig 1:**
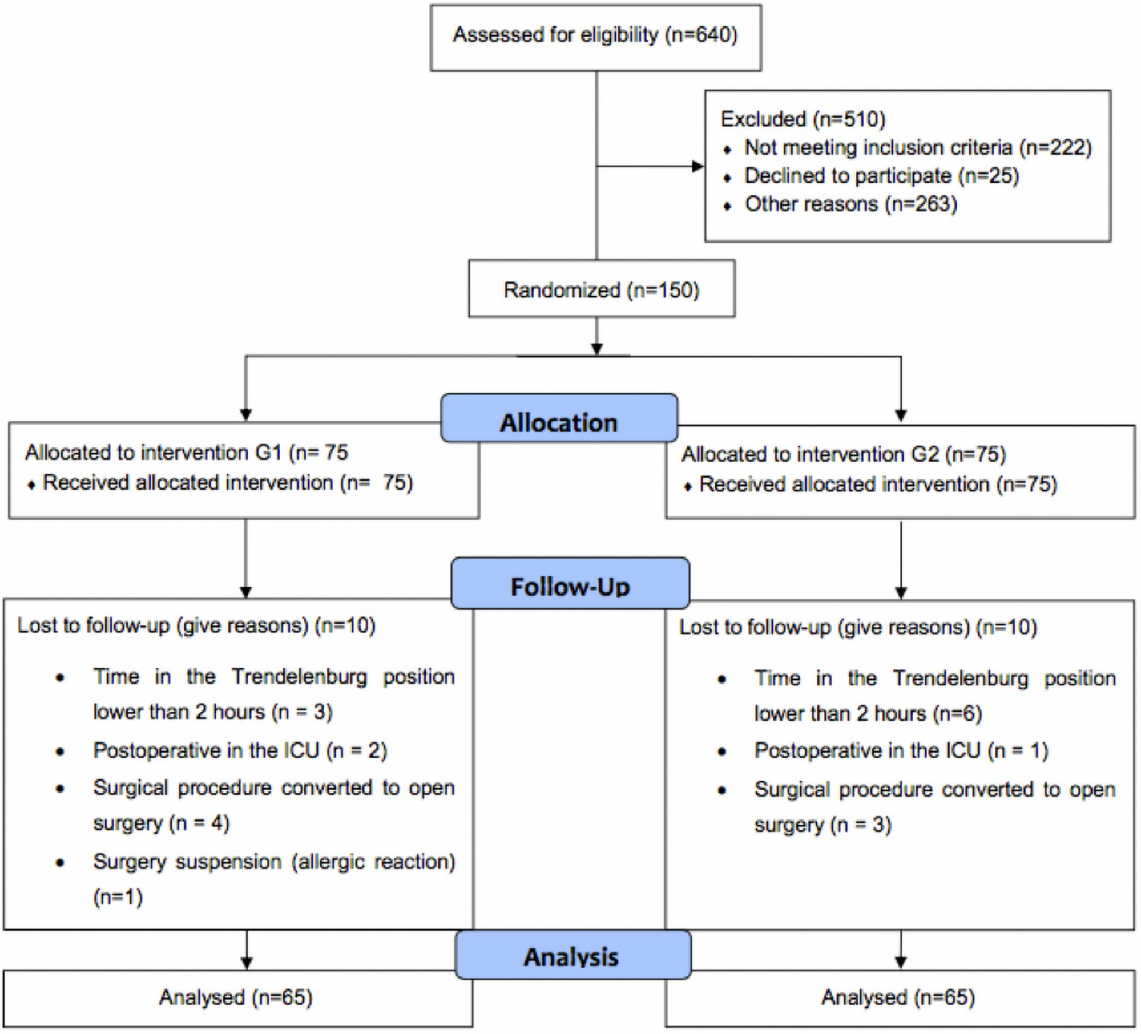
Flowchart of the study.

Eligible participants were oncology patients from the A.C.Camargo Cancer Center, Brazil, who were older than 18 years and had an American Society of Anesthesiologists (ASA) classification < 3. These participants were submitted to elective video laparoscopic surgery in the TP while remaining in this position for a minimum of 2 hours (Fig 1).

Exclusion criteria included absolute contraindications related to the SB, difficult airway prediction, previous NCD, chronic use of benzodiazepines, anemia (hemoglobin < 10 mg/dl), kidney disease (stage > G3a), body mass index (BMI) > 30 Kg/m^2^, contraindications associated with the maintenance of desflurane sedation, and patients who did not sign the informed consent form (Fig 1).

Patient follow-up was considered interrupted if the required Trendelenburg time was not reached, there was a need for postoperative time in the intensive care unit, the surgical procedure was converted to open surgery, a difficult airway was not foreseen, the SB failed, or surgery suspension for any reason.

### Primary and secondary endpoints

Our primary objective was to analyze whether the anesthetic techniques used for oncology video laparoscopic surgeries performed in the TP were associated with different incidences of delirium during the postoperative period until the patient’s hospital discharge.

Our secondary objective was to analyze whether other factors (sex, attendance, education, age, ASA classification, surgery type, Trendelenburg angle and time, pneumoperitoneum pressure, morphine dose, time in the post-anesthesia care unit [PACU], length of hospital stay, BMI, and desflurane, remifentanil, and vasopressor doses) were associated with the onset of delirium.

### Conducting the study

Patients were allocated to 2 groups: the general anesthesia (GA) group and the general anesthesia plus SB (GSA) group. Sequential allocation was used to control patient age (> 65 years) as a possible confounding factor for the outcome of interest [23], and was performed via R version 3.5 (R Foundation for Statistical Computing, Vienna, Austria).

The following patient parameters were monitored: the electrocardiogram, non-invasive pressure, pulse oximetry, capnogram, bispectral index (BIS), level of the neuromuscular block using accelerometry (TOF-Watch^®^SX; Organon), esophageal temperature, and Trendelenburg angle.

All patients received midazolam 0.03 mg/kg (IV) and 500 ml of crystalloid solution (IV) before the block, together with 4 ml/kg/hour of crystalloid solution plus volume, depending on clinical parameters during the surgical procedure.

Spinal procedures were performed under an aseptic technique (chlorhexidine gluconate) in the sitting position via an L3/L4 puncture with a 27-G Whitacre needle and desired sensory level block T6. SB was performed with hyperbaric bupivacaine 0.5% (15 mg). Both groups received intrathecal morphine (50 mcg). General anesthesia was induced with fentanyl 3 mcg/kg (IV), propofol 2 mg/kg (IV), and rocuronium 0.6 mg/kg (IV). Patients were ventilated with an oxygen and air mixture with a 40% oxygen inspired fraction (FI) while maintaining the expired carbon dioxide concentration between 35 and 45 mmHg. Anesthesia was maintained with remifentanil (ng/ml) and desflurane.

Sedation was guided by maintaining the BIS between 40 and 60, and the remifentanil dose was adjusted based on vital signs. Neuromuscular blockade was monitored with a train-of-four monitor through the orbicularis oculi muscle. When necessary, the dose used was one-third of the rocuronium dose used during induction. Patients with a mean arterial pressure (MAP) lower than 60 mmHg were medicated with vasopressors (metaraminol/ephedrine) according to their heart rate.

Patients received (IV): dexamethasone 4 mg, ondansetron 8 mg, metamizole 2 g and parecoxib 40 mg, provided they had no contraindications and were kept warm with thermal blankets.

After surgery, the neuromuscular blockade was antagonized with sugammadex based on the train-of-four.

The patient was evaluated constantly for any signs of cognitive alterations, from the moment of awakening from anesthesia to the moment of hospital discharge. Delirium assessments were conducted using the Confusion Assessment Method (CAM) [24] (Table 1) and Richmond Agitation-Sedation Scale RASS [25] (Table 2) by the following people at the following times: the anesthetist attending to the patient during the operation, the PACU-trained nurse during the PACU stay, and the researcher during the postoperative time. Delirium was defined as a positive CAM at any one of these times.

**Table 1:** Confusion Assessment Method (CAM).

**1. Acute onset and fluctuating course**	
Is there evidence of an acute change in mental status from the patient’s baseline?	YES/NO
**2. Inattention**	
Did the patient have difficulty focusing attention, for example, being easily distractible or having difficulty keeping track of what was being said?	YES/NO
**3. Disorganized thinking**	
Was the patient’s thinking disorganized or incoherent, such as rambling or irrelevant conversation, unclear or illogical flow of ideas, or unpredictable switching from subject to subject?	YES/NO
**4. Altered level of consciousness**	
Did the patient have a change in the level of consciousness, such as lethargy, stupor, or coma?	YES/NO

* *The diagnosis of delirium requires the presence of answer yes in 1 AND 2 plus either 3 OR 4. Adapted: INOUYE et al. 1990.

**Table 2:** Richmond agitation–sedation scale.

Score	Term	Description
+ 4	Combative	Overtly combative or violent; immediate danger to staff
+ 3	Very agitation	Pulls on or removes tube(s) or catheter(s) or has aggressive behavior toward staff
+ 2	Agitated	Frequent nonpurposeful movement or patient–ventilator dyssynchrony
+ 1	Restless	Anxious or apprehensive but movements not aggressive or vigorous
0	Alert and calm	
- 1	Drowsy	Not fully alert, but has sustained (more than 10 seconds) awakening, with eye contact, to voice
- 2	Light sedation	Briefly (less than 10 seconds) awakens with eye contact to voice
- 3	Moderate sedation	Any movement (but no eye contact) to voice
- 4	Deep sedation	No response to voice, but any movement to physical stimulation
- 5	Unarousable	No response to voice or physical stimulation

Adapted: SESSLER et al. 2002.

Pain levels were evaluated using a numeric rating scale (NRS). Those who exhibited pain with an NRS score > 4 were administered morphine 1 mg every 10 minutes. The researcher evaluated the postoperative pain daily until the moment of hospital discharge.

### Measurements and data handling

Variables studied included: the presence or absence of delirium during the postoperative period, age, sex, ASA classification, BMI, presence or absence of higher education (college degree), postoperative pain, MAP, intraoperative heart rate, pneumoperitoneum pressure, Trendelenburg angle and time, length of stay after surgery, and the desflurane (FE%), remifentanil (ng/ml), vasopressor, and PACU morphine doses.

### Sample size calculation

Due to the lack of previous studies involving the specific group of patients used in this study, a literature search was performed for similar studies [26]. Accordingly, the proportion of patients with delirium was fixed as GA 0.05 and GSA 0.23. The sample size was calculated based on these proportions, considering the results of a two-proportion test. The test power was set at 0.85 with a significance level of 0.05. The sample size calculated for each group was 65 patients.

### Statistical analyses

A descriptive analysis was performed to assess the clinical and demographic characteristics of the two anesthesia groups associated with the presence or absence of delirium during the postoperative period. Summary measures of position and dispersion, such as the mean / standard deviation and medians / interquartile ranges, are reported for quantitative variables, and the absolute and relative frequencies (%) are reported for qualitative variables.

Univariate and multiple logistic regressions were used to identify the variables associated with delirium. Odds ratios (ORs) and 95% confidence intervals (CIs) were calculated for all variables. A stepwise selection algorithm was then applied, with different significance levels associated for entry (p < 0.10) and retention (0.05). Variables were removed from the model if they were insignificant and did not act as confounders (change in β coefficient > 20%). The assumption of linearity was assessed for all continuous variables. Overall performance, calibration, and discriminatory power of the final multiple logistic regression model were assessed using the Brier score, Hosmer–Lemeshow goodness-of-fit test, area under the curve, and receiver operating characteristic curves [27, 28].

In order to assess the effects of group variables (GA and GSA) and time on hemodynamic parameters (MAP and heart rate), the nonparametric technique of variance analysis (ANOVA) with repeated measures was applied to the data. This technique is based on the concept of the “relative effect of treatments” that was proposed by Brunner, Munzel, and Puri (1999) [27].

Statistical analyses were performed using R software version 3.5 (R Foundation for Statistical Computing, Vienna, Austria). The significance level was fixed at 5% for all tests.

## Results

The surgical and clinical characteristics of the study population are presented in Table 3. The incidence of delirium during the postoperative period was significantly higher in the GA group (30.8%) than in the GSA group (13.8%), with p = 0.035 (Table 3). During the postoperative period, delirium developed in 22.3% patients as follows: delirium developed and subsided in the operating room in 76% patients (16 patients in the GA group and 6 patients in the GSA group), delirium developed in the operating room and subsided during post-anesthetic recovery in 21% patients (3 patients in the GA group and 3 patients in the GSA group), and delirium developed and ceased during the first postoperative day in 1 patient in the GA group. All cases were classified as hyperactive delirium.

**Table 3:** Clinical and demographic characteristics according to group.

Variable		GA (n = 65)	GSA (n = 65)	p
**Prior Surgery**				
**Sex**—n (%)				
Female		15 (23.1)	24 (36.9)	0.126*
Male		50 (76.9)	41 (63.1)	
**Higher education**—n (%)				
Yes		28 (43.1)	29 (44.6)	0.999*
No		37 (56.9)	36 (55.4)	
**Elderly (> 65 years)**—n (%)				
Yes		18 (27.7)	18 (27.7)	0.999*
No		47 (72.3)	47 (72.3)	
**ASA (American Society of Anesthesiologists classification)–n (%)**				
1		13 (20.0)	11 (16.9)	0.821*
2		52 (80.0)	54 (83.1)	
**Surgery type—**n (%)				
Urology		40 (61.5)	33 (51.0)	0.373*
Pelvic		17 (26.0)	19 (29.0)	
Gynecology		8 (12.5)	13 (20.0)	
**Age (years)**	^a^	57 (48–65.5)	59 (49.5–66)	0.563**
	^b^	56.1 ± 11.8	57.37 ± 11.4	
**During Surgery**				
**Trendelenburg position angle (degrees)**	^a^	22 (18.5–25)	21 (19–26)	0.974**
	^b^	22.0 ± 5.0	22.0 ± 5.5	
**Time in the Trendelenburg position (minutes)**	^a^	135 (120–180)	180 (135–210)	0.002**
	^b^	150.8 ± 43.2	176 ± 48.6	
**Pneumoperitoneum pressure (mmHg)**	^a^	13 (12–14)	12 (12–14)	0.174**
	^b^	13.1 ± 1.7	12.7 ± 1.4	
**Dose of morphine in the PACU (mg)**	^a^	2 (0–3)	0 (0–2)	0.001**
	^b^	1.6 ± 1.5	0.6 ± 1.0	
**PACU time (minutes)**	^a^	90 (60–120)	90 (60–120)	0.416**
	^b^	96.4 ± 39.1	90.9 ± 38.2	
**Hospitalization days**	^a^	2 (1–3)	2 (1–3)	0.606**
	^b^	2.4 ± 1.3	2.5 ± 1.6	
**BMI (kg/m2)**	^a^	26 (24–28)	27 (25–29)	0.724**
	^b^	26.3 ± 2.7	26.5 ± 2.7	
**Desflurane (EF%)**	^a^	5.1 (4.7–6)	4.8 (4–5)	0.001**
	^b^	5.1 ± 0.6	4.7 ± 0.7	
**Remifentanil (mcg/kg/min)**	^a^	0.08 (0.05–0.12)	0 (0–0.05)	0.001**
	^b^	0.08 ± 0.08	0.03 ± 0.05	
**Vasopressor (bolus)**	^a^	0 (0–2)	3 (1–5)	0.001**
	^b^	1.5 ± 2.5	3.2 ± 2.6	
**Surgery time (minutes)**	^a^	195 (180–240)	240 (195–270)	0.002**
	^b^	210.8 ± 43.2	236.5 ± 48.6	

^a^ Median (1°Q–3°Q);

^b^ Mean ± SD;

* Chi-squared test;

** Student t-Test;

Abbreviations: SD–standard deviation; PACU—post-anesthesia care unit; BMI–body mass index; ASA—American Society of Anesthesiologists; GA—general anesthesia; GSA—general anesthesia plus spinal block; 1°Q–first quartile; 3°Q–third quartile.

According to the results obtained in Table 3, there is no evidence of association between the group and the variables, indicating that the groups are balanced in relation to the randomization variable.

Patients in the GA group were administered a higher concentration of desflurane, higher dose of remifentanil, smaller number of bolus doses of vasopressor, and higher dose of morphine during the postoperative period (p < 0.001) (Table 3).

**Fig 2:**
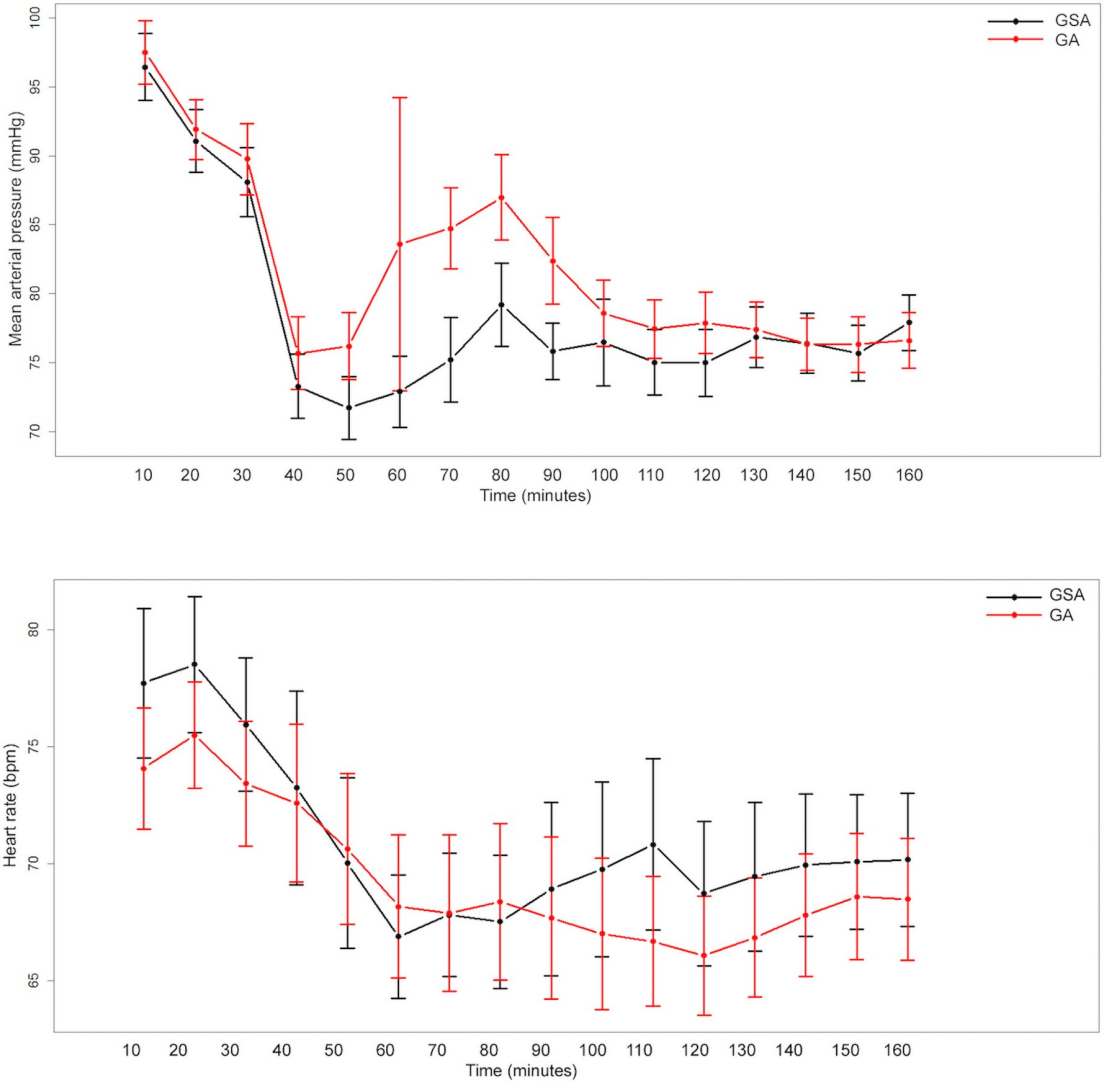
Hemodynamic parameters evaluated over time in both groups of patients. GA—general anesthesia; GSA—general anesthesia plus spinal block.

For MAP, a significant group effect (p = 0.006), time effect (p <0.0001), and interaction between group and time (p = 0.0001) were observed. For heart rate, there was no evidence of a group effect (p = 0.379), but there was an effect of time (p <0.0001). That is, for MAP, there was a significant difference between groups and over time (effect of time); in contrast, for heart rate, there was a difference only over time, regardless of the group (Fig 2).

Risk factors for the occurrence of delirium were assessed for the entire sample, using logistic regression (Tables 4 and 5). Initially, each isolated group was evaluated, and then both groups combined were analyzed. Only the multiple model for the entire sample underwent adjustment because the number of patients within each group was too small for a multiple model.

**Table 4:** Univariate logistic regression models of delirium outcome.

Variable	Category	OR (95% CI)	p
**ASA**			
	1	Ref	
	2	1.112 (0.376–3.292)	0.848
**Anesthesia**			
	GSA	Ref	
	GA	2.765 (1.148–6.661)	0.023
**BMI**			
	Normal	Ref	
	Overweight	0.359 (0.155–0.838)	0.018
**Specialty**			
	Urology	Ref	
	Pelvic	0.374 (0.128–1.089)	0.071
	Gynecology	0.244 (0.052–1.139)	0.073
**Higher education**			
	No	6.901 (2.240–21.265)	0.001
	Yes	Ref	
**Sex**			
	Male	3.314 (1.068–10.283)	0.038
	Female	Ref	
**Elderly (> 65 years)**			
	Yes	4.081 (1.706–9.768)	0.002
	No	Ref	
**BMI**	Unit	0.853 (0.730–0.997)	0.045
**Surgery time**	Unit	0.996 (0.987–1.006)	0.446
**Desflurane**	Unit	2.310 (1.242–4.298)	0.008
**Vapressor**	Unit	0.855 (0.712–1.027)	0.095
**Age**	Unit	1.043 (1.002–1.086)	0.041
**Trendelenburg angle**	Unit	1.040 (0.961. 1.127)	0.326
**Trendelenburg time**	Unit	0.996 (0.987. 1.006)	0.446
**Pneumoperitoneum pressure**	Unit	1.663 (1.202. 2.301)	0.002
**Time PACU**	Unit	0.996 (0.986. 1.008)	0.559
**Morphine PACU**	Unit	1.871 (1.363. 2.569)	< 0.001
**Hospitalization days**	Unit	0.777 (0.561. 1.077)	0.130
**Extubation pain**	Unit	0.947 (0.778. 1.153)	0.589

*OR—odds ratio (estimated from the logistic regression model); CI—confidence interval of the estimated OR; PACU—post-anesthesia care unit; BMI—body mass index; NA—not applicable; ASA—American Society of Anesthesiologists; GA—general anesthesia; GSA—general anesthesia plus spinal block.

**Table 5:** Parameters estimates from multiple logistic regression models of delirium outcome.

Variable	Category	OR (95% CI)	p
**Anesthesia**			
	GSA	Ref	
	GA	3.409 (1.210–9.602)	0.020
**Higher education**			
	Yes	Ref	
	No	6.233 (1.800–21.579)	0.003
**Elderly (> 65 years)**			
	No	Ref	
	Yes	3.383 (1.242–9.211)	0.017
**Pneumoperitoneum pressure**	Unit	1.703 (1.149–2.523)	0.008

*OR—odds ratio (estimated from the logistic regression model); CI—confidence interval of the estimated OR; GA—general anesthesia; GSA—general anesthesia plus spinal block.

The desflurane, remifentanil, vasopressor bolus, and morphine doses were not used in our logistic regression model because these factors are dependent on the anesthesia type.

**Fig 3:**
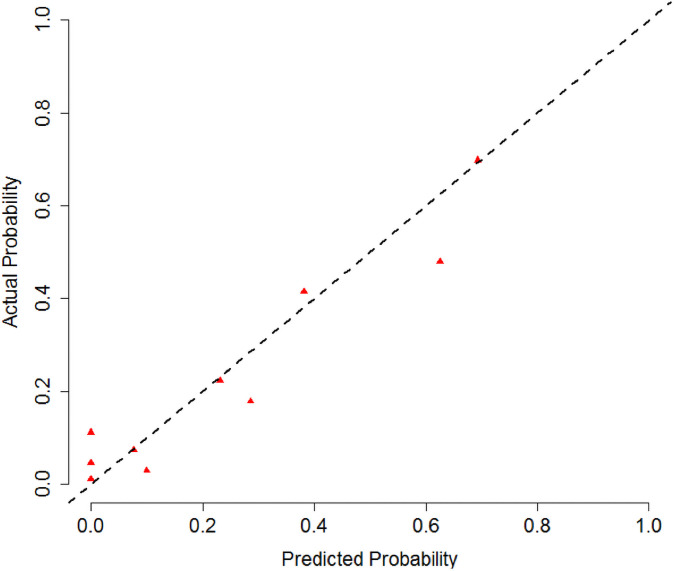
Calibration plot for the delirium model.

**Fig 4:**
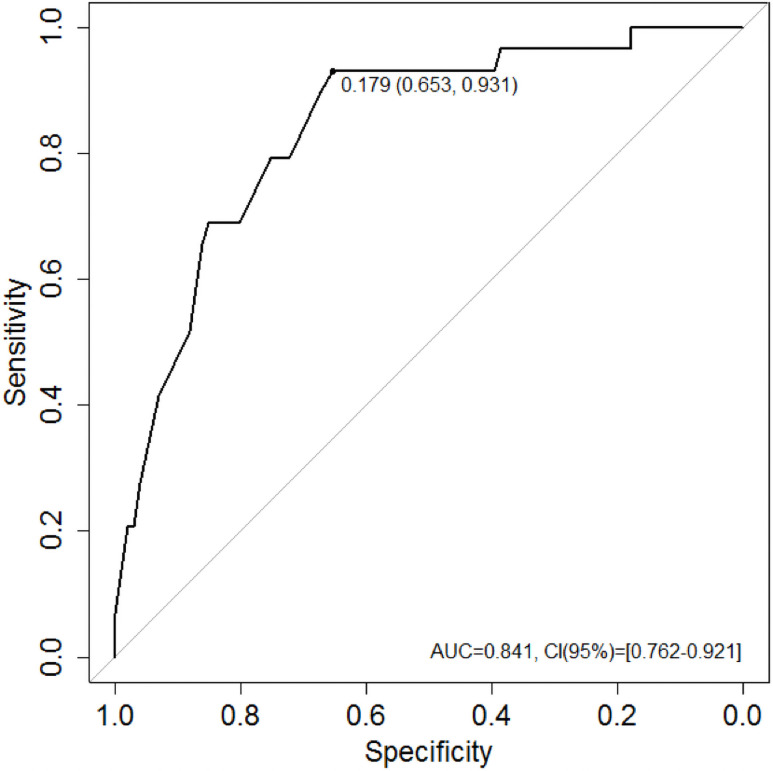
A receiver operating characteristics (ROC) curve obtained via the multiple logistic regression modelling. AUC, area under the curve; CI, confidence interval.

The multiple logistic regression models revealed that in the entire sample, GA (OR: 3.4, 95% CI 1.2–9.6 p = 0.020), absence of higher education (OR: 6.2, 95% CI 1.8–21.5 p = 0.003), advanced age (OR: 3.3, 95% CI 1.2–9.2 p = 0.017), and increased pneumoperitoneum pressure (OR: 1.7, 95% CI 1.1–2.5 p = 0.008) were associated with increased delirium incidence during the postoperative period (Table 5). Overall performance (Brier score 0.123), calibration (Hosmer-Lemeshow test: χ^2^ (8) = 6.374; p = 0.606) (Fig 3), and discriminatory power (AUC 0.841, 95% CI 0.762–0.921) were adequate (Fig 4).

## Discussion

In this trial, oncology patients undergoing laparoscopic surgeries under general anesthesia associated with a SB in the TP exhibited a lower incidence of delirium during the postoperative period until hospital discharge. Absence of higher education, advanced age and increased pneumoperitoneum pressure were identified as risk factors associated with an increased incidence of delirium during the postoperative period.

Previous studies have shown that the TP causes physiological changes because the head is in a downward position, increasing the intracranial pressure, alone or in combination with pneumoperitoneum [29]. Several studies have attempted to relate the TP to the development of delirium during the postoperative period [13, 30, 31]. This relationship was not observed in our study.

In the previous literature on anesthetic techniques influencing the incidence of delirium in the postoperative period, no significant differences were found among various anesthetic techniques, such as general anesthesia [32–36], SB [8, 32–34, 36], combined anesthesia [33], peripheral nerve blockades [32], and mild/deep sedation [32]. The current literature provides no recommendations regarding the anesthetic technique to be used in order to prevent delirium in the postoperative period [32]. Our findings suggest that the anesthetic technique can influence the incidence of delirium in the postoperative period.

Pain during the postoperative period is an important recognized risk factor for delirium [6]. With a neuraxial blockade, it is possible to have better pain control during the postoperative period [19, 20, 37], and to also have lower anesthetic agent dose consumption [20, 22], with analgesia lasting up to 24 hours [38]. The incidence of emergence delirium was lower in the GSA group than in the GA group, probably due to residual analgesia from the SB.

Hemodynamic changes caused by a SB have been exhaustively studied. Among patients submitted to this procedure, hypotension can occur at an incidence of up to 50%, and bradycardia can appear with an incidence of around 15%; both are the most common complications and result from the sympathetic blockade, yet both are easily reversed with hydration or vasopressors [39]. These data were also validated in the present study results; we observed an important reduction in blood pressure and a greater need for vasopressors in the GSA group than in the GA group. While several studies have uncovered a relationship between intraoperative hypotension and the development of delirium during the postoperative period [32], this relationship was not observed in our study, probably due to the use of vasopressors to minimize the blood pressure decrease.

While physiological changes related to pneumoperitoneum insufflation, such as increased intracranial pressure and altered cerebral blood flow, have already been described as risk factors for postoperative delirium [40, 41], pneumoperitoneum pressure itself has not. In the present study, increased pneumoperitoneum pressure was an independent risk factor associated with the development of delirium during the postoperative period. Therefore, we can speculate the existence of a potential ideal pneumoperitoneum pressure for delirium prevention during the postoperative period.

In addition, there are several other factors associated with an increased incidence of delirium in the postoperative period. Elderly patients have a recognized higher risk of developing delirium during the postoperative period [3]. Increased anesthetic depth has been associated with development of PD. Therefore, avoiding low BIS values, associated with high doses of anesthetic agents, is recommended [3, 32]. Absence of higher education is also associated with higher delirium incidence during the postoperative period [10, 42], possibly caused by difficulties in understanding the information provided by the surgical team.

### Limitations

This study involved patients from a single institution, rendering external validation challenging; further, the small sample size limits the assessment of secondary objectives.

Criteria for defining an NCD during the perioperative period by the DSM-5 and revised nomenclature consensus are clear. However, the application of this classification in clinical practice is difficult in certain situations, mainly due to the differentiation between emergence delirium and PD, since this difference is subtle and occurs at frequently overlapping time intervals.

### Strengths

Our study was prospective and randomized, with high levels of involvement and training of the professionals included. Our results contribute to a better understanding of the risk factors for NCDs, and protection techniques for issues that may become apparent during the perioperative period.

## Conclusions

In this trial, oncology patients undergoing laparoscopic surgeries under general anesthesia associated with a SB in the TP exhibited a lower incidence of delirium during the postoperative period until hospital discharge.

Absence of higher education, advanced age and increased pneumoperitoneum pressure were identified as risk factors associated with an increased incidence of delirium during the postoperative period.

Our findings will prompt further studies to evaluate the relationship between anesthesia and delirium.

## Supporting information

S1 File (PDF)

S2 File (PDF)

S1 Checklist CONSORT 2010 checklist of information to include when reporting a randomised trial*. (PDF)

S1 Protocol (DOCX)
