# Supplementary material for: Spinal block and delirium in oncologic patients after laparoscopic surgery in the Trendelenburg position: A randomized controlled trial
Source: PLoS One. 2021 May 17;16(5):e0249808. doi: 10.1371/journal.pone.0249808 (PMC8128254; doi:10.1371/journal.pone.0249808)
Supplement: S1 File — (PDF) [file pone.0249808.s001.pdf]

ANÁLISE DE DUAS TÉCNICAS ANESTÉSICAS NO APARECIMENTO DO DELIRIUM PÓS OPERATÓRIO DE PACIENTES ONCOLÓGICOS SUBMETIDOS A CIRURGIAS DE VIDEOLAPAROSCOPIA EM POSICÃO DE TRENDELEMBURG

JORGE KIYOSHI MITSUNAGA JUNIOR

Dissertação apresentada à Fundação Antônio Prudente para obtenção do título de Doutor em Ciências

Área de Concentração: Oncologia

Orientadora: Dra. Giane Nakamura

São Paulo
2017

1 INTRODUÇÃO

Há tempos o câncer tem enorme impacto em nossa sociedade e com isso vem sendo tema importante em todas as pautas de discussões.

Segundo estimativas mundiais da Organização Mundial da Saúde (OMS), foram detectados 14 milhões de casos novos de câncer e um total de 8,2 milhões de óbitos por câncer, em todo o mundo, em 2012. Estes números se tornam alarmantes e poderão se tornar ainda maiores se amplas medidas preventivas não forem realizadas (Ministério da Saúde 2016).

No que se refere ao Brasil, a estimativa para o ano de 2016/2017 aponta a ocorrência de cerca de 600 mil casos novos de câncer. Excetuando-se o câncer de pele não melanoma (aproximadamente 180 mil casos novos), ocorrerão cerca de 420 mil casos novos de câncer (Ministério da Saúde 2016).

O arcabouço normativo da área da oncologia no Sistema Único de Saúde (SUS) tem como base duas portarias e nelas se consideram que para cada 1000 casos novos de câncer espera-se que 500 a 600 necessitem de cirurgia oncológica, 700 de quimioterapia e 600 de radioterapia. Em 2010, foram realizadas 44.580 cirurgias oncológicas somente no estado de São Paulo (CECILIOII e TAMELINIII 2011).

Cirurgias estas que em sua imensa maioria são de grande porte envolvendo grande ressecções, sujeitas a um grau elevado de dor no pós-operatório e consequentemente acarretam um período prolongado de recuperação. Com relação aos pacientes, estes têm varias comorbidades e já

passaram por vários procedimentos e tratamentos, em especial quimioterapia e radioterapia (VERONESI e STAFYLA 2012).

Na era dos procedimentos minimamente invasivos, a cirurgia videolaparoscópica vem ganhando unanimidade (HENNY e HOFLAND 2005). Dentre suas inúmeras vantagens estão: menor dor no pós-operatório, melhores resultados estéticos devido a pequenas incisões, rápido retorno às atividades diárias, redução nos dias de internação, menor sangramento intra-operatório, menos complicações pulmonares no pós-operatório, menos infecção de ferida operatória, menor desarranjo metabólico no pós-operatório e consequentemente redução de custos (GERGES et al. 2006).

Esta técnica consiste na criação do pneumoperitôneo, que é a insuflação de um gás através de uma pequena incisão na parede abdominal com o objetivo de separá-la dos órgãos internos possibilitando uma melhor visibilidade da área a ser tratada pelo cirurgião e manipulação dos instrumentos dentro da cavidade. (BIRCH DW, DANG JT, SWITZER NJ, MANOUCHEHRI N, SHI X, HADI G 2016).

A pressão do pneumoperitôneo é definida como normal e adequada quando os valores se encontram entre 12 a 15 mmHg, sendo considerada baixa quando os valores se encontram entre 5 a 7 mmHg, abaixo desta última faixa ficaria praticamente impossível a pratica desta técnica cirúrgica, pois a visualização das estruturas ficaria muito prejudicada (NEUDECKER et al. 2002). O aumento desta pressão acarreta aumento da absorção do gás, eleva o diafragma diminuindo a complacência pulmonar e aumentando a pressão de via aérea. Aumento da pressão intra-abdominal aumenta o retorno venoso devido a compressão da vasculatura esplâncnica. Pneumoperitôneo também

aumenta a resistência vascular sistêmica e resistência vascular pulmonar. Durante a fase inicial do pneumoperitôneo, o débito cardíaco é reduzido por diminuição do retorno venoso. Embora estas alterações sejam bem toleradas por indivíduos saudáveis com boa reserva cardiopulmonar, pacientes sem esta reserva podem não tolerar tão bem (GURUSAMY KS, VAUGHAN J 2014). Aproximadamente 17% dos pacientes submetidos a colecistectomia videolaparoscopica são ASA III ou IV (GIGER et al. 2006), já em cirurgias oncológicas este número pode ser ainda maior.

O gás ideal seria aquele com as seguintes propriedades: mínima absorção peritoneal, mínimo efeitos fisiológicos, rápida excreção de qualquer gás absorvido, não ser comburente, mínimo efeito de embolização intravascular e alta solubilidade sanguínea (GERGES et al. 2006).

Dentre os gases disponíveis, o mais comumente utilizado é o dióxido de carbono por possuir características mais próximas do gás ideal, mas o mesmo também possui características indesejadas (GERGES et al. 2006). Devido a sua característica de difusibilidade o pneumoperitonêo causado pelo dióxido de carbono é desfeito rapidamente, porém apesar da rapidez, uma certa quantidade pode permanecer e causar desconforto. Devido sua alta solubilidade o mesmo pode ocasionar hipercarbia, hipercapnia e acidose (MENES e SPIVAK 2000).

Assim que ocorre o estabelecimento do pneumoperitôneo há um aumento da pressão intra-abdominal e consequentemente efeitos cardiovasculares, respiratórios e neurológicos (CONACHER et al. 2004;HENNY e HOFLAND 2005;GERGES et al. 2006;KALMAR et al. 2010).

A gravidade dos possíveis efeitos cardiovasculares como variação de pressão arterial, arritimias e parada cardíaca estão relacionados com o volume de dióxido de carbono absorvido, volume intravascular do paciente, técnica ventilatória, condições cirúrgicas, agentes anestésicos utilizados, porém os fatores mais importantes são pressão intra-abdominal e posição do paciente (HENNY e HOFLAND 2005; GERGES et al. 2006).

Os efeitos respiratórios incluem redução de volume pulmonar, aumento de pressão de via aérea, diminuição de complacência pulmonar secundária ao aumento de pressão intra-abdominal e posição do paciente (CONACHER et al. 2004;HENNY e HOFLAND 2005;GERGES et al. 2006;KALMAR et al. 2010).

As alterações neurológicas surgem devido ao aumento de pressão intracraniana, diminuição de perfusão cerebral, hipercapnia e aumento de resistência vascular sistêmica (CONACHER et al. 2004;GERGES et al. 2006;KALMAR et al. 2010).

No que se refere ao posicionamento do paciente para cirurgias videolaparoscópicas são descritas as mais diversas, porém uma das mais desafiadoras e com mais alterações fisiológicas ocorre nas que necessitam serem realizadas em posição de Trendelenburg (MARTIN JT 1997).

Cefalodeclive ou mais conhecida como posição de Trendelenburg foi descrita em meados do século 19, por um cirurgião alemão Friedrich Trendelenburg, continua sendo utilizada rotineiramente nos dias atuais, principalmente em cirurgias do trato geniturinário ou colorretais. Uma das grandes vantagens do uso desta posição é a melhora da exposição do campo cirúrgico provendo a melhora da técnica cirúrgica (MARTIN 1997;TALAB et al. 2016). Pacientes com duração prolongada nesta posição podem ter edema de

face, conjuntiva, laringe e língua com potencial risco de obstrução de via área. Cuidado especial também deve ser dado com o posicionamento do paciente, já que o mesmo se torna sujeito a lesão de plexo nervoso (ANDREA CESTARI e MARIA BUFFI, EMANUELE SCAPATICCI, GIOVANNI LUGHEZZANI, ANDREA SALONIA, ALBERTO BRIGANTI, PATRIZIO RIGATTI, FRANCESCO MONTORSI 2010) (MICHEL W. COPPIETERS 2002).

Algumas desvantagens deste procedimento associado a posição de Trendelenburg seriam a alterações fisiopatológicas pulmonares com formação de atelectasias e aumento de pressão de via aérea. Também estão associados com aumento de pressão intracraniana e formação de edema cerebral. Estes efeitos poderiam levar a baixa perfusão cerebral e potencial desemparelhamento de suprimento de oxigênio para o cérebro (CONACHER et al. 2004;CLOSHEN et al. 2014;ROSENDAL et al. 2014).

Para cirurgias videolaparoscópicas a anestesia geral com agentes de curta duração é quase unânime. No que se refere a técnica para manutenção da anestesia geral, atualmente existem duas técnicas: anestesia venosa total e anestesia inalatória. Ambas são universalmente utilizadas, com raras exceções de uma técnica ser mais vantajosa perante à outra técnica como em determinadas situações especificas. Anestesia venosa total seria melhor indicada para pacientes com histórico de náuseas/vômito no pós operatório (APFEL et al. 2002; LEE et al. 2015), não é agente desencadeador de hipertermia maligna (HOPKINS 2000; MANI e MORTON 2010) menos distúrbios de comportamento no pós operatório de cirurgias pediátrica (ORTIZ et al. 2014), anestesia em neurocirurgia com tendência a hipertensão intracraniana (PETERSEN et al. 2003). Anestesia inalatória seria mais

benéfica com neuroproteção (KITANO et al. 2007), cardioproteção (FRABDORF et al. 2009; PS 2010) e menor chance de recall no intra-operatório (AMERICAN SOCIETY OF ANESTHESIOLOGISTS TASK FORCE ON INTRAOPERATIVE AWARENESS 2006).

A associação com raquianestesia, desde que seguidas as orientações de suas contraindicações (QUADRO 1), também é vista como vantajosa, já que produz despertar mais precoce, diminui náuseas e vômitos, dor pós-operatória, tempo de internação hospitalar, custo efetivo, melhora satisfação do paciente (BESSA et al. 2012; CARD et al. 2015; DAY et al. 2015; GERGES et al. 2006; GHOSH et al. 2015; SINHA et al. 2009; WONGYINGSINN et al. 2012) e principalmente a diminuição do consumo dos agentes anestésicos da anestesia geral (GERGES et al. 2006).

Quadro 1 – Contraindicações à raquianestesia.

Absolutas	***Relativas***
Recusa do paciente	Coagulopatia
Hipovolemia significativa	Sepse/bacteremia
Infecção no sítio de punção	Doença neurológica prévia
Hipertensão intracraniana	Neuropatia periférica
Coagulopatia significativa	Esclerose múltipla
	Processo desmielinizante

Adaptado: (NEAL et al. 2015)

Como desvantagem, ao associar as duas técnicas anestésicas, os pacientes tornam-se susceptíveis aos eventos adversos inerentes à raquianestesia. Dentre esses, os mais comuns são hipotensão em torno de 5% (CARPENTER, RL; CAPLAN , RA;BROWN 1992; HARTMANN et al. 2002),

náuseas e vômitos descritas em 15% (BORGEAT et al. 2003), prurido varia de 30 a 100 % dos pacientes (RATHMELL et al. 2005), retenção urinária foi observada em até 35% dos pacientes (KUIPERS et al. 2004) , tremores, que podem ocorrer em até 50% dos pacientes (CROWLEY e BUGGY 2008)(CROWLEY e BUGGY 2008). Complicações intrínsecas ao procedimento são raras, mas descritas na literatura tais como hematoma, infecção e colaplso cardiovascular fatal em 1/100.000 pacientes (COOK et al. 2009; MOEN et al. 2004).

Outra possibilidade seria a raquianestesia somente com opióide, mais comumente a morfina (ARAIMO MORSELLI et al. 2016; DICHTWALD et al. 2016), trazendo o benefício de uma analgesia por um período de 12 a 24 horas, sem as consequências cardiovasculares causado pelo bloqueio simpático como consequência do uso do anestésico local (ARAIMO MORSELLI et al. 2016).

Atualmente, em cirurgias videolaparoscópicas, não está claro qual a melhor técnica anestésica para tal procedimento. Existem tentativas de buscar indicações da raquianestesia através de revisões sistemáticas da literatura (PROSPECT [s.d.]) ou guidelines (AMERICAN SOCIETY OF ANESTHESIOLOGISTS TASK FORCE ON ACUTE PAIN MANAGEMENT 2012), porém nenhum é categórico e com forte nível de evidencia em afirmar tal conduta.

No pós-operatório, uma das alterações neurológicas mais temidas é o delirium, sendo considerado a complicação pós-cirúrgica mais comum, ocorrendo entre 5 a 50% dos procedimentos (INOUYE et al. 2014). O mesmo é um perverso e complicado processo que traz inúmeros desafios para a

equipe que acompanha o paciente (ALDECOA et al. 2017; STEINER 2011a). Especialmente deletério nos idosos, já que um único episódio pode desencadear uma cascata de eventos como: hospitalização prolongada, perda de independência funcional, redução da função cognitiva e morte (SACZYNSKI JS, MARCANTONIO ER, QUACH L, FONG TG, GROSS A, INOUYE SK 2012). Algumas metanálises sugerem que um único episódio de delirium aumenta em 2 vezes o risco de óbito, aumenta o tempo de ventilação mecânica, tempo de UTI e de internação médica (MCDANIEL e BRUNEY 2012).

Delirium é definido no DSM V (*Diagnostic and Statiscal Manual of Mental Disorders*) como: distúrbio de consciência, alteração de cognição e tempo de curso flutuante e agudo (AMERICAN PSYCHIATRIC ASSOCIATION 2013).

Clinicamente, para diagnóstico de delirium existem vários métodos, pórem o mais utilizado é realizado por meio do *Confusion Assemenment Method* (CAM) (FABBRI et al. 2001; STEINER 2011b)(ANEXO 1), um simples método de rastreio baseado em quatro perguntas que tem uma sensibilidade de 86% e especificidade de 93% (WONG et al. 2010). A lista de verificação para diagnóstico de delírio é composto por 4 itens: 1 – Início agudo; 2- Distúrbio de atenção; 3- Pensamento desorganizado; 4 – Alteração do nível de consciência. O diagnóstico de delírio requer a presença dos critérios 1 e 2 mais os critérios 3 ou 4.

Uma variante conhecida como CAM-ICU é usada com frequência em paciente intubados ou sedados (ELY et al. 2001; LUETZ et al. 2010), porém na atualidade ainda não existe um método específico e bem estabelecido para avaliação do delirium de pacientes cirúrgicos em pós-operatório imediato.

Existem 3 subtipos de delirium: hiperativo (agitação e inquietação) (25%), hipoativo (letargia e desatenção) (50%) e misto (25%). Em um estudo envolvendo 400 pacientes, com idade mediana de 57 (44,67) anos, avaliados na sala de recuperação anestésica, 124 (31%) tiveram delirium na admissão na sala de recuperação anestésica, 59 (15%) com 30 minutos após admissão, 32 (8%) com 60 minutos após admissão e 15 (4%) na alta da sala de recuperação anestésica. Nos pacientes com sinais de delirium, sinais hipoativos estavam presentes em 56% na admissão da sala de recuperação anestésica e em 92% durante a estadia na sala de recuperação anestésica (CARD et al. 2015). O subtipo hipoativo é o mais prevalente e também associado com maior mortalidade. Embora não exista um consenso sobre a definição de delirium pós-operatório, a maioria dos estudos define sua ocorrência entre 24 a 72 horas pós-operatório, entretanto alguns ainda ocorram após a sua alta hospitalar (WHITLOCK et al. 2011;CARD et al. 2015).

A escala de Richmond Agitation Sedation Scale (RASS), desenvolvida com objetivo de caracterizar o nível de consciência e agitação, é rotineiramente utilizada em associação ao CAM para caracterizar o delirium. Sinais de delirium hiperativo são definidos com pontuação RASS indo de +1 (paciente ansioso) até +4 (paciente agitado) acompanhado de CAM positivo. Sinais de delirium hipoativo serão definidos como RASS indo de – 5 (paciente não responde) até 0 (paciente calmo ou sonolento) acompanhado de CAM negativo (NASSAR et al. 2008; SESSLER et al. 2002)(ANEXO 2).

Atualmente, a hipótese mais aceita para a fisiopatologia do delirium no pós-operatório, parece ser um insulto neurológico agudo em um paciente com um substrato suscetível. Tal disjunção resulta de alteração no balanço de

neurotransmissores ou mediadores neuro inflamatórios no cérebro, resultando em sintomas clínicos de delirium. As vias mais comumente afetadas incluem acetilcolina, dopamina, acido gama aminobutírico (GABA) e serotonina, onde o marcador neuro inflamatório mais comum envolve o eixo hipófise hipotalâmica, representado pelo cortisol, assim como proteína C reativa (PCR), pro calcitonina, fator de necrose tumoral e varias interleucinas como IL-6, IL-8 e IL-10 (ALI et al. 2011). Embora haja aceitação geral de quais indicadores clínicos definam o substrato susceptível, tem ocorrido aumento nos esforços para identificar os marcadores bioquímicos pré-operatórios e pós-operatórios que possam ajudar a identificar os potenciais riscos para delirium baseados nas teorias citadas (MCDANIEL e BRUNEY 2012).

Os fatores de riscos presumidos são multifatoriais e estes podem ser agrupados em não modificáveis e modificáveis. No primeiro grupo se encontram o aumento da idade, mal estado funcional já detectado no pré-operatório, comorbidades (demência, depressão, nefropatia, cardiopatia e doenças pulmonares), tipo de cirurgia (emergência, ortopedia, cardiovascular) (SCHOEN et al. 2011). Já no segundo grupo incluem certas classes de medicamentos (opióide, benzodiazepínico, anti-histamínico, dihidropiridina) (CLEGG e YOUNG 2011), polifarmácia (mais que seis medicamentos ou a adição de três novos), infecção ou inflamação, dor, distúrbio eletrolítico (sódio e potássio), alterações hematológicas (anemia, hipoxemia) , alteração do ciclo sono vigília (WHITLOCK et al. 2011; SANDERS et al. 2011; CHAPUT e BRYSON 2012), pressão de p
erfusão intraoperatória e possivelmente profundidade do plano anestésico (SIEBER et al. 2010).

No que se refere ao uso fármacos, os opióides devem ser prescritos com precaução, já que seu uso, principalmente em altas doses poderiam aumentar o risco de delirium. Por outro lado, dores severas não tratadas adequadamente estão associadas ao surgimento do mesmo. Já com relação aos benzodiazepínicos, a associação se dá principalmente com doses altas e com fármacos de longa duração (CLEGG e YOUNG 2011).

É de conhecimento geral que uma avaliação pré-operatória e identificação dos fatores de risco reduz muito as chances do evento (ADULTS 2014).

Uma das maneiras encontradas para tentar diminuir a incidência de delirium no intraoperatório é através da modernização da monitorização. Em relação à monitorização multimodal (débito cardíaco, profundidade de hipnose, oxigenação cerebral), esta vem ganhando bastante foco. Ao detectar e reduzir as complicações, contribui para que a videolaparoscopia venha a se aproximar do método de cirurgia ideal (GREEN et al. 2014).

Neuromonitorização durante procedimento anestésico pode melhorar o despertar do paciente (GUARRACINO 2008;FEDOROW e GROCOTT 2010). O BIS (índice Bi-Espectral) é derivado de uma análise do eletroencefalograma (EEG) e representa a profundidade anestésica por meio de um único valor que pode variar de 0 a 100 e que se correlaciona com a sedação e hipnose, sendo usado clinicamente para titular os agentes anestésicos. Valores entre 40 e 60 são referidos como adequados para a cirurgia (AG et al. 2016) . O mesmo foi um dos primeiros índices de EEG e se tornou um dos monitores mais utilizados com este intuito no mundo. Por meio dele é possível realizar uma hipnose nos níveis adequados para a realização do procedimento (PUNJASAWADWONG

et al. 2010;KLOPMAN e SEBEL 2011). Hipnose guiada pelo BIS poderia influenciar no nível cognitivo e mortalidade pós-operatório (FARAG et al. 2006; MONK et al. 2005), entretanto ainda é controverso (KERTAI et al. 2011; LESLIE e SHORT 2011).

Hoje muito se comenta sobre o Triple Low, onde pacientes com baixo Bispectral Index Scores (BIS) com baixa concentração de agentes anestésicos associado a baixa pressão de perfusão apresenta elevadas taxas de morbimortalidade em 30 a 90 dias (DEINER STACIE AND JEFFREY H. 2013).

2 JUSTIFICATIVA

No âmbito internacional, o número de cirurgias videolaparoscópicas vem aumentando gradativamente (DANIEL J. RISKIN, MICHAEL T. LONGAKER, MICHAEL GERTNER 2006; KAVIC 1998) e dentre as técnicas anestésicas disponíveis, a anestesia geral e a anestesia geral associada com raquianestesia, estão entre as mais utilizadas (GERGES et al. 2006; HENNY e HOFLAND 2005).

Devido a alterações fisiológicas ocasionadas pelo pneumoperitôneo (HENNY e HOFLAND 2005; KALMAR et al. 2010) associadas com a posição de Trendelenburg (CLOSHEN et al. 2014; KALMAR et al. 2010), especula-se o aumento na incidência de delirium no pós-operatório destas cirurgias.

Desta forma devido a inexistência de trabalhos publicados sobre a incidência de delirium, no pós-operatório em cirurgias videolaparoscópicas oncológicas em posição de Trendelenburg, foi optado pela realização do mesmo.

3 OBJETIVOS

O objetivo deste estudo foi analisar se as técnicas anestésicas, empregadas para cirurgias videolaparoscópicas oncológicas em posição de Trendelenburg, diferiam com relação a incidência de delirium no pós-operatório.

4 MATERIAL E MÉTODOS

Estudo prospectivo e randomizado, no qual serão incluídos pacientes provenientes do centro cirúrgico do Hospital A.C. Camargo, com 18 anos ou mais, ASA inferior a 3, submetidos a cirurgia eletiva laparoscópica em posição de Trendelenburg com duração de no mínimo de 2 horas na posição de cefalodeclive e que concordarem em assinar o termo de consentimento livre e esclarecido.

Estudo será avaliado pelo comitê de ética e pesquisa (CEP) da Fundação Antônio Prudente (FAP).

Pacientes serão alocados de forma sequencial em possíveis 2 grupos. A alocação sequencial (FOSSALUZA et al. 2009) será utilizada a fim de controlar algumas variáveis confundidoras. Envelopes opacos serão gerados e posteriormente abertos pelo coordenador da anestesia do centro cirúrgico antes da cirurgia daquele respectivo dia.

Serão excluídos os seguintes pacientes:

1. Não desejarem participar da pesquisa.
2. Contraindicação absolutas e relativas à raquianestesia (QUADRO 1).
3. Previsão via aérea difícil (possibilidade de intubação acordado) (QUADRO 2).
4. Diagnóstico prévio de: distúrbio cognitivo e/ou depressão.
5. Uso crônico de benzodiazepínicos (uso durante as ultimas 12 semanas) (NEUROLOGIA 2016).
6. Encaminhados para realizar o pós-operatório em UTI.

7. Anemia (Hemoglobina < 10).
8. Infecção atual.
9. Doença renal com estagio > G3a (taxa de filtração glomerular < 45 ml/min/1.73 m^2) (FOUNDATION [s.d.]).
10. Índice de massa corpórea (IMC) caracterizada por obesidade (IMC > 30 Kg/m^2).
11. História de náuseas/vômitos em procedimentos anestésicos prévios.
12. Hipertermia maligna.

Quadro 2 – Preditores Via Aérea Difícil

Incisivos superiores longos
Relação entre os incisivos maxilares/mandibulares durante o fechamento normal da mandíbula (retrognata)
Relação entre os incisivos maxilares/mandibulares durante a protrusão voluntária da mandíbula (inabilidade protrusão da mandíbula)
Pequena abertura da boca (distancia entre os incisivos < 3 cm)
Mallampati III e IV
Arco do palato arqueado
Perda da complacência do espaço mandibular (rígido, endurecido, ocupado por massa)
Distância tireomento (< 3 dedos)
Pescoço curto
Pescoço grosso``
Mobilidade cervical limitada

Modificado: (APFELBAUM JL, HAGBERG CA 2013)

Esses pacientes foram monitorizados no intraoperatório com ECG, pressão não invasiva, oximetria de pulso, capnografia, termômetro, BIS e monitor de bloqueador neuromuscular.

Os pacientes serão dividos em 2 grupos:

1) anestesia balanceada + raquianestesia com morfina 50 mcg
2) anestesia balanceada + raquianestesia com 2,5 ml bupivacaína pesada 0,5% + morfina 50 mcg

Todos os pacientes receberão em sala operatória a medicação pré-anestésica endovenosa midazolan 0,03 mg/kg para conforto do mesmo e 500ml de solução cristalóide antes da indução/bloqueio associado a 4 ml/kg/hora de solução cristalóide mais volume a depender de parâmetros clínicos.

A raquianestesia será realizada na posição sentada, antissepsia/assepsia com clorexidine alcoólico, localização do espaço L3/L4, punção com agulha whitacre 27 G, barbotagem de 0,5 ml e injeção com duração de 5 a 7 segundos

Na sequência será realizada anestesia geral com pré-oxigenação 8 litros/minuto durante 5 minutos + fentanil 3 mcg/kg + propofol 2 mg/ kg + rocurônio 0,6 mg/kg. A anestesia será mantida com remifentanil (mcg/kg/min) e desflurano (Fe%).

Hipnose será guiada mantendo BIS entre 40 e 60 e a dose de remifentanil será guiada pelos sinais vitais.

Em ambos os grupos, os pacientes serão mantidos aquecidos com manta térmica e terão sua temperatura esofagiana mensurada (distância de 40 cm dos dentes incisivos) (DANIEI 2008; WANG 2016).

Os seguintes parâmetros serão mensurados: frequência cardíaca, pressão arterial, BIS, capnografia, saturação, TOF, temperatura esofagiana e ângulo do Trendelenburg.

Os momentos mensurados serão entrada do paciente em sala

operatória, após medicação pré-anestésica, pré-indução anestésica, após indução anestésica, após incisão cirúrgica, após insuflação pneumoperitôneo, após posição de Trendelenburg, de 15 em 15 minutos até o final do procedimento e após extubação.

Finalizado o procedimento cirúrgico e 5 minutos após extubação, os sinais vitais (pressão arterial, frequência cardíaca, saturação) serão coletados e o paciente será avaliado com relação à presença de delirium. Será realizado dipirona 2 gramas e parecoxibe 40 mg não havendo contraindicação, para analgesia de pós-operatório,

Na sequencia os pacientes serão encaminhados para a sala de recuperação anestésica (SRPA), onde continuarão sendo monitorizados com ECG, pressão não invasiva e oxímetros de pulso. Serão ainda avaliados com relação a dor na escala de estimativa numérica (NRS) (0= sem dor, até 10 = pior dor), na chegada e de 30 em 30 minutos até o momento de alta para o quarto. Aqueles que apresentarem dor na escala de estimativa numérica (NRS) > 4 na SRPA, serão tratados com morfina 1 mg a cada 10 min ou NRS <4. Aqueles que apresentarem náuseas/vômitos, serão medicados com alizaprida 50 mg.

Os pacientes serão avaliados, com relação ao aparecimento de delirium por meio do *Confusion Assemenment Method* (CAM) (ANEXO 1) em associação será utilizado a escala de Richmond Agitation Sedation Scale (RASS) (ANEXO 2).

Essa avaliação será realizada por enfermeiras, previamente treinadas, na chegada a SRPA, de 30 em 30 minutos até o momento da alta da sala de recuperação pós-anestésica e de 24 horas em 24 horas até o momento da alta

hospitalar, pelo próprio pesquisador. Nos casos em que o paciente apresentar episódio de delirium persiste por mais de 1 hora será solicitado a avaliação de um psiquiatra para acompanhamento e tratamento.

No que se refere a análise estatística, devido a inexistência de estudos envolvendo este grupo específico de pacientes, foi realizada uma simulação com a incidência de delirium vista por Sanders et al 2011.

- Proporção de casos entre os expostos: 5%
- Proporção de casos entre os não expostos: 21%
- Risco relativo calculado: 0,2381
- Nível de significância: 5%
- Poder do teste: 85%
- Teste de hipótese: monocaudal
- Tamanho da amostra calculado para cada grupo: 62

Considerando perdas, foi estipulada o número de pacientes para cada grupo (ANEXO 3).

- Grupo 1 – "n:65"- anestesia balanceada + raquianestesia com + morfina 50 mcg.
- Grupo 2 – "n:65"- anestesia balanceada + raquianestesia com 2,5 ml bupivacaína pesada 0,5% + morfina 50 mcg.

Inicialmente será realizada uma análise descritiva dos dados, em que a distribuição de frequência absoluta/relativa serão apresentadas para as variáveis qualitativas e as principais medidas-resumo, como média, mediana, máximo, mínimo e desvio padrão serão apresentadas para as variáveis quantitativas. Com o objetivo de avaliar uma possível associação entre as variáveis de interesse com o desfecho (delirium e não delirium), o teste de

independência (qui-quadrado ou exato de Fisher) será aplicado. Além disso, a fim de avaliar e quantificar o impacto de cada variável independente no desfecho de interesse, o modelo de regressão logística simples e múltipla serão ajustado aos dados. Em todos os testes será fixado um nível de significância de 5%. As análises serão realizadas utilizando o software SPSS versão 23 e o software livre R versão 3.4

5 RESULTADOS

Projeto em andamento.

6 DISCUSSÃO

Projeto em andamento.

7 CONCLUSÃO

Projeto em andamento.

8 REFERÊNCIAS BIBLIOGRÁFICAS

Adults TAGSEP ON PD IN O. Postoperative Delirium in Older Adults: Best Practice Statement from the American Geriatrics Society. **Journal of the American College of Surgeons** 2014;

Ag M, Wang M, Mj W, et al. Anaesthetic interventions for prevention of awareness during surgery (Review) SUMMARY OF FINDINGS FOR THE MAIN COMPARISON. **Cochrane Library** 2016;

Aldecoa C, Bettelli G, Bilotta F, et al. European Society of Anaesthesiology evidence-based and consensus-based guidelines on postoperative delirium. **European Journal of Anaesthesiology** 2017; 34:192–214.

Ali S, Patel M, Jabeen S, et al. Insight into delirium. **Innovations in Clinical Neuroscience** 2011; 8:25–34.

American Psychiatric Association. **Diagnostic and Statistical Manual of Mental Disorders (DSM-V)**. [s.l: s.n.].

American Society of Anesthesiologists Task Force on Acute Pain Management. Practice guidelines for acute pain management in the perioperative setting: an updated report by the American Society of Anesthesiologists Task Force on Acute Pain Management. **Anesthesiology** 2012; 116:248–73.

American Society of Anesthesiologists Task Force on Intraoperative Awareness. Practice Advisory for Intraoperative Awareness and Brain Function Monitoring. **Anesthesiology** 2006; 104:847–864.

Andrea Cestari N, Maria Buffi, Emanuele Scapaticci, Giovanni Lughezzani, Andrea Salonia, Alberto Briganti, Patrizio Rigatti, Francesco Montorsi GG. Simplifying Patient Positioning and Port Placement During Robotic-Assisted Laparoscopic Prostatectomy. **EUROPEAN UROLOGY** 2010; 57:530–533.

Apfel CC, Kranke P, Katz MH, et al. Volatile anaesthetics may be the main cause of early but not delayed postoperative vomiting: A randomized controlled

trial of factorial design. **British Journal of Anaesthesia** 2002; 88:659–668.

Apfelbaum JL, Hagberg CA CR. Practice guidelines for management of the difficult airway:an updated report by theAmerican Society of Anesthesiologists Task Force on Management of the Difficult Airway. **Anesthesiology** 2013; 118:251–270.

Araimo Morselli FSM, Zuccarini F, Caporlingua F, et al. Intrathecal Versus Intravenous Morphine in Minimally Invasive Posterior Lumbar Fusion: A Blinded Randomized Comparative Prospective Study. **Spine** 2016; 42:281–284.

Bessa SS, Katri KM, Abdel-Salam WN, El-Kayal E-SA, Tawfik TA. Spinal Versus General Anesthesia for Day-Case Laparoscopic Cholecystectomy: A Prospective Randomized Study. **Journal of Laparoendoscopic & Advanced Surgical Techniques** 2012; 22:550–555.

Birch DW, Dang JT, Switzer NJ, Manouchehri N, Shi X, Hadi G KS. Heated insufflation with or without humidification for laparoscopic abdominal surgery (Review). **Cochrane Library** 2016;

Borgeat A, Ekatodramis G, Schenker C. Postoperative nausea and vomiting in regional anesthesia: a review. **Anesthesiology** 2003; 530–547.

Card E, Pandharipande P, Tomes C, et al. Emergence from general anaesthesia and evolution of delirium signs in the post-anaesthesia care unit. **British Journal of Anaesthesia** 2015; 115:411–417.

Carpenter, RL; Caplan , RA;Brown D. Incidence and risk factors for side effects of spinal anesthesia. **Anesthesiology** 1992; 76:906–916.

CecilioII MCMMACMNFMAM, TameliniII RM. Diretrizes para a atenção oncológica no Estado de São Paulo: contribuições para o debate. **BEPA** 2011; 8:24–43.

Chaput AJ, Bryson GL. Postoperative delirium: risk factors and management: continuing professional development. **Canadian journal of anaesthesia = Journal canadien d'anesthésie** 2012; 59:304–20.

Clegg A, Young JB. Which medications to avoid in people at risk of delirium: A systematic review. **Age and Ageing** 2011; 40:23–29.

Closhen D, Treiber A-H, Berres M, et al. Robotic assisted prostatic surgery in the Trendelenburg position does not impair cerebral oxygenation measured using two different monitors: A clinical observational study. **European journal of anaesthesiology** 2014; 31:104–9.

Conacher ID, Soomro NA, Rix D. Anaesthesia for laparoscopic urological surgery. **British Journal of Anaesthesia** 2004; 93:859–864.

Cook TM, Counsell D, Wildsmith JAW. Major complications of central neuraxial block: Report on the Third National Audit Project of the Royal College of Anaesthetists. **British Journal of Anaesthesia** 2009; 102:179–190.

Crowley LJ, Buggy DJ. Shivering and Neuraxial Anesthesia. **Regional Anesthesia and Pain Medicine** 2008; 33:241–252.

Daniel S. Temperature Monitoring and Perioperative Thermoregulation. **Anesthesiology** 2008; 109:318–38.

Daniel J. Riskin, Michael T. Longaker, Michael Gertner TMK. Innovation in Surgery. **Annals of Surgery** 2006; 244:686–693.

Day AR, Smith RVP, Scott MJP, Fawcett WJ, Rockall TA. Randomized clinical trial investigating the stress response from two different methods of analgesia after laparoscopic colorectal surgery. **British Journal of Surgery** 2015; 102:1473–1479.

Deiner Stacie and Jeffrey H. Long-Term Outcomes in Elderly Surgical Patients. **Mt Sinai J of Medicine of Medicine** 2013; 79:95–106.

Dichtwald S, Ben-Haim M, Papismedov L, Hazan S, Cattan A, Matot I. Intrathecal morphine versus intravenous opioid administration to impact postoperative analgesia in hepato-pancreatic surgery: a randomized controlled trial. **Journal of Anesthesia** 2016; 1–9.

Ely EW, Ely EW, Inouye SK, et al. Delirium in Mechanically Ventilated Patients. **Jama** 2001; 286:2703–2710.

Erman AB, Collar RM, Griffith KA, et al. Sentinel lymph node biopsy is accurate and prognostic in head and neck melanoma. **Cancer** 2012; 118:1040–1047.

Fabbri RMA, Moreira MA, Garrido R, Almeida OP. Validity and reliability of the portuguese version of the confusion assessment method (CAM) for the detection of delirium in the elderly. **Arquivos de Neuro-Psiquiatria** 2001; 59:175–179.

Farag E, Chelune GJ, Schubert A, Mascha EJ. Is depth of anesthesia, as assessed by the Bispectral Index, related to postoperative cognitive dysfunction and recovery? **Anesthesia and Analgesia** 2006; 103:633–640.

Fedorow C, Grocott HP. Cerebral monitoring to optimize outcomes after cardiac surgery. **Current opinion in anaesthesiology** 2010; 23:89–94.

Fossaluza V, Diniz JB, De Bragança Pereira B, Miguel EC, De Bragança Pereira CA. Sequential Allocation to Balance Prognostic Factors in a Psychiatric Clinical Trial. **Clinics (Sao Paulo, Brazil)** 2009; 64:511–518.

Foundation NK. **CLINICAL PRACTICE GUIDELINES For Chronic Kidney Disease: Evaluation, Classification and Stratification**. [s.l: s.n.].

Frabdorf J, De Hert S, Schlack W. Anaesthesia and myocardial ischaemia/reperfusion injury. **British Journal of Anaesthesia** 2009; 103:89–98.

Gerges FJ, Kanazi GE, Jabbour-Khoury SI. Anesthesia for laparoscopy: A review. **Journal of Clinical Anesthesia** 2006; 18:67–78.

Ghosh S, Saha S, Mallik S, Pal S, Das W, Bhattacharya S. Comparison between general anesthesia and spinal anesthesia in attenuation of stress response in laparoscopic cholecystectomy: A randomized prospective trial. **Saudi Journal of Anaesthesia** 2015; 9:184.

Giger UF, Michel J, Opitz I, Inderbitzin DT. Risk Factors for Perioperative Complications in Patients Undergoing Laparoscopic Cholecystectomy : Analysis of 22 , 953 Consecutive Cases from the Swiss Association of Laparoscopic and Thoracoscopic Surgery Database. **Journal of American**

College of Surgeons 2006; 723–728.

Green D, Bidd H, Rashid H. Multimodal intraoperative monitoring: An observational case series in high risk patients undergoing major peripheral vascular surgery. **Int J Surg** 2014; 12:231–236.

Guarracino F. Cerebral monitoring during cardiovascular surgery. **Current opinion in anaesthesiology** 2008; 21:50–54.

Gurusamy KS, Vaughan J DB. Low pressure versus standard pressure pneumoperitoneum in laparoscopic cholecystectomy (Review). **Cochrane Library** 2014;

Hartmann B, Junger A, Klasen J, et al. The Incidence and Risk Factors for Hypotension After Spinal Anesthesia Induction: An Analysis with Automated Data Collection. **Anesthesia and analgesia** 2002; 94:1521–1529.

Henny CP, Hofland J. Laparoscopic surgery: Pitfalls due to anesthesia, positioning, and pneumoperitoneum. **Surgical Endoscopy and Other Interventional Techniques** 2005; 19:1163–1171.

Hopkins PM. Malignant hyperthermia: advances in clinical management and diagnosis. **British journal of anaesthesia** 2000; 85:118–128.

Inouye SK, Westendorp RGJ, Saczynski JS. Delirium in elderly people. **The Lancet** 2014; 383:911–922.

Inouye SK, Van Dick CH, Alessi CA BS. Clarifying confusion: the confusion assessment method: a new method for detection of deliruium. **Ann Intern Med** 1990; 133:941–8.

Kalmar AF, Foubert L, Hendrickx JFA, et al. Influence of steep Trendelenburg position and CO2 pneumoperitoneum on cardiovascular, cerebrovascular, and respiratory homeostasis during robotic prostatectomy. **British Journal of Anaesthesia** 2010; 104:433–439.

Kavic MS. A decade of laparoscopic cholecystectomy. **JSLS : Journal of the Society of Laparoendoscopic Surgeons / Society of Laparoendoscopic Surgeons** 1998; 2:319–20.

Kertai MD, Palanca BJ A, Pal N, et al. Bispectral index monitoring, duration of bispectral index below 45, patient risk factors, and intermediate-term mortality after noncardiac surgery in the B-Unaware Trial. **Anesthesiology** 2011; 114:545–556.

Kitano H, Kirsch JR, Hurn PD, Murphy SJ. Inhalational anesthetics as neuroprotectants or chemical preconditioning agents in ischemic brain. **Journal of cerebral blood flow and metabolism : official journal of the International Society of Cerebral Blood Flow and Metabolism** 2007; 27:1108–28.

Klopman MA, Sebel PS. Cost-effectiveness of bispectral index monitoring. **Curr Opin Anaesthesiol** 2011; 24:177–181.

Kuipers PW, Kamphuis ET, Van Venrooij GE, et al. Intrathecal opioids and lower urinary tract function: a urodynamic evaluation. **Anesthesiology** 2004; 100:1497–1503.

Lee WK, Kim MS, Kang SW, Kim S, Lee JR. Type of anaesthesia and patient quality of recovery: A randomized trial comparing propofol-remifentanil total i.v. anaesthesia with desflurane anaesthesia. **British Journal of Anaesthesia** 2015; 114:663–668.

Leslie K, Short TG. Low bispectral index values and death: The unresolved causality dilemma. **Anesthesia and Analgesia** 2011; 113:660–663.

Luetz A, Heymann A, Radtke FM, et al. Different assessment tools for intensive care unit delirium: which score to use? **Critical care medicine** 2010; 38:409–418.

Mani V, Morton NS. Overview of total intravenous anesthesia in children. **Paediatric Anaesthesia** 2010; 20:211–222.

Martin JT WM. **Positioning in Anesthesia and Surgery**. Philadelphia: WB Saunders, 1997.

McDaniel M, Bruney C. Postoperative delirium: etiology and management. **Curr Opin Crit Care** 2012; 18:372–6.

Menes T, Spivak H. Laparoscopy Searching for the proper insufflation gas.

Surgical Endoscopy 2000; 1050–1056.

MICHEL W. COPPIETERS MVDVAKHS. Positioning in Anesthesiology: Toward a Better Understanding of Stretch-Induced Perioperative Neuropathies. **Anesthesiology** 2002;

Ministério da Saúde. Estimativa/2016 incidência de câncer no Brasil. **Ministério da Saúde** 2016;

Moen V, Dahlgren N, Irestedt L. Severe Neurological Complications after Central Neuraxial Blockades in Sweden 1990 –1999. **Anesthesiology** 2004; 101:950–9.

Monk TG, Saini V, Weldon BC, Sigl JC. Anesthetic management and one-year mortality after noncardiac surgery. **Anesthesia and Analgesia** 2005; 100:4–10.

Nassar AP, Neto RCP, De Figueiredo WB, Park M. Validity, reliability and applicability of Portuguese versions of sedation-agitation scales among critically ill patients. **Sao Paulo Medical Journal** 2008; 126:215–219.

Neal JM, Barrington MJ, Brull R, et al. The Second ASRA Practice Advisory on Neurologic Complications Associated With Regional Anesthesia and Pain Medicine Executive Summary 2015. 2015; 40:401–430.

Neudecker J, Sauerland S, Neugebauer E, et al. The European Association for Endoscopic Surgery clinical practice guideline on the pneumoperitoneum for laparoscopic surgery. **Surgical Endoscopy and Other Interventional Techniques** 2002; 16:1121–1143.

Neurologia AB DE PAB DE. Abuso e Dependência de Benzodiazepínicos. **Diretrizes AMB** 2016;

Ortiz AC, Atallah AN, Matos D, Da Silva EM. Intravenous versus inhalational anaesthesia for paediatric outpatient surgery. **The Cochrane database of systematic reviews** 2014; 2:CD009015.

Petersen KD, Landsfeldt U, Cold GE, et al. Intracranial pressure and cerebral hemodynamic in patients with cerebral tumors: A randomized prospective study

of patients subjected to craniotomy in propofol-fentanyl, isoflurane-fentanyl, or sevoflurane-fentanyl anesthesia. **Anesthesiology** 2003; 98:329–336.

Prospect. **Procedure Specific Postoperative Pain Management**. Disponível em: <http://www.postoppain.org>. Acesso em: 15 abr. 2017.

PS P. Cardioprotection by noble gases. **J Cardiothorac Vasc Anesth** 2010; 24:143–163.

Punjasawadwong Y, Phongchiewboon A, Bunchungmongkol N. Bispectral index for improving anaesthetic delivery and postoperative recovery (Review) Bispectral index for improving anaesthetic delivery and postoperative recovery. **Cochrane Library** 2010; 10–12.

Rathmell JP, Lair TR, Nauman B. The role of intrathecal drugs in the treatment of acute pain. **Anesthesia and analgesia** 2005; 101:S30–S43.

Rosendal C, Markin S, Hien MD, Motsch J, Roggenbach J. Cardiac and hemodynamic consequences during capnoperitoneum and steep Trendelenburg positioning: Lessons learned from robot-assisted laparoscopic prostatectomy. **Journal of Clinical Anesthesia** 2014; 26:383–389.

Saczynski JS, Marcantonio ER, Quach L, Fong TG, Gross A, Inouye SK JR. Cognitive Trajectories after Postoperative Delirium. **The New England journal of M edicine** 2012; 5:30–39.

Sanders RD, Pandharipande PP, Davidson AJ, Ma D, Maze M. Anticipating and managing postoperative delirium and cognitive decline in adults. **BMJ (Clinical research ed.)** 2011; 343:d4331.

Schoen J, Meyerrose J, Paarmann H, Heringlake M, Hueppe M, Berger K-U. Preoperative regional cerebral oxygen saturation is a predictor of postoperative delirium in on-pump cardiac surgery patients: a prospective observational trial. **Critical Care** 2011; 15:R218.

Sessler CN, Gosnell MS, Grap MJ, et al. The Richmond Agitation – Sedation Scale Validity and Reliability in Adult Intensive Care Unit Patients. **Am J Respir Crit Care Med** 2002;

Sieber FE, Zakriya KJ, Gottschalk A, et al. Sedation depth during spinal anesthesia and the development of postoperative delirium in elderly patients undergoing hip fracture repair. **Mayo Clinic proceedings. Mayo Clinic** 2010; 85:18–26.

Sinha R, Gurwara AK, Gupta SC. Laparoscopic Cholecystectomy Under Spinal Anesthesia: A Study of 3492 Patients. **Journal of Laparoendoscopic & Advanced Surgical Techniques** 2009; 19:323–327.

Steiner LA. Postoperative delirium. Part 1: pathophysiology and risk factors. **European Journal of Anaesthesiology** 2011a; 28:628–636.

Steiner LA. Postoperative delirium. Part 2: detection, prevention and treatment. **European Journal of Anaesthesiology** 2011b; 28:723–732.

Talab SS, Elmi A, Sarma J, Barrisford GW, Tabatabaei S. Safety and Effectiveness of SAF-R, a Novel Patient Positioning Device for Robot-Assisted Pelvic Surgery in Trendelenburg Position. **Journal of endourology / Endourological Society** 2016; 30:286–92.

Veronesi U, Stafyla V. Grand challenges in surgical oncology. 2012; 2:1–3.

Wang M. Optimal Depth for Nasopharyngeal Temperature Probe Positioning. **Anesthesia and Analgesia** 2016;

Whitlock E, Vannucci A, Avidan M. Postoperative delirium. **Minerva Anaesthesiology** 2011; 77:448–456.

Wong CL, Holroyd-Leduc J, Simel DL, Straus SE. Does this patient have delirium?value of bedside instruments. **JAMA** 2010; 304:779.

Wongyingsinn M, Baldini G, Stein B, Charlebois P, Liberman S, Carli F. Spinal analgesia for laparoscopic colonic resection using an enhanced recovery after surgery programme: Better analgesia, but no benefits on postoperative recovery: A randomized controlled trial. **British Journal of Anaesthesia** 2012; 108:850–856.

Anexo 1 - Confusion Assement Method - CAM

**Confusion Assement Method - CAM**	
**1. Inicio Agudo**	
Há evidências de mudança aguda do estado mental de base do paciente?	((SIM) (NÃO)
**2. Distúrbio de atenção**	
O paciente teve dificuldade em focalizar sua atenção: por exemplo, distraiu-se facilmente ou teve dificuldade em acompanhar o que estava sendo dito?	((SIM) (NÃO)
**3. Pensamento desorganizado**	
O pensamento do paciente era desorganizado ou incoerente, com a conversão dispersiva ou irrelevante, fluxo de ideias pouco claro ou ilógico, ou mudança imprevisível do assunto?	((SIM) (NÃO)
**4. Alteração no nível de consciência**	
O paciente apresenta alteração do nível de consciência como letárgico, torporoso, comatoso?	((SIM) (NÃO)

Devem estar presentes os critérios 1 e 2 mais 3 ou 4

Adaptado de: (INOUYE SK, VAN DICK CH, ALESSI CA 1990)

Anexo 2 - Richmond Agitation Sedation Scale - RASS

Richmond Agitation Sedation Scale - RASS

Score	***Termo***	***Descrição***
+4	Agressivo	Muito agressivo, violento, perigo imediato à equipe.
+3	Muito agitado	Puxa ou retira tubos e cateteres; agressivo.
+ 2	Agitado	Movimentos não intencionais incoordenados frequentes, luta contra a ventilação.
+1	Inquieto	Ansioso, mas sem movimentos agressivos ou vigorosos.
0	Alerta e calmo	Alerta e calmo.
-1	Sonolento	Não se encontra completamente alerta, mas tem o despertar sustentado (abertura ocular / contato visual) ao som da voz (> 10 segundos).
-2	Sedação leve	Desperta brevemente e faz contato visual ao som da voz (<10 segundos).
-3	Sedação moderada	Movimentos ou abertura ocular ao som da voz (mas sem contato visual).
- 4	Sedação profunda	Sem resposta ao som da voz, mas movimenta-se ou abre os olhos com estimulação física.
- 5	Não despertável	Sem resposta ao som da voz ou estimulação física.

Fonte: Adaptado de: (SESSLER et al. 2002).

4 MATERIAL AND METHODS

Prospective, randomized study including patients from the operating room of AC Camargo Hospital, aged 18 years or older, ASA less than 3, undergoing laparoscopic elective surgery in a Trendelenburg position lasting at least 2 hours in the cephalodeclive and agree to sign the free and informed consent form.

Study will be evaluated by the ethics and research committee (CEP) of the Antônio Prudente Foundation (FAP).

Patients will be sequentially allocated to possible 2 groups. Sequential allocation (FOSSALUZA et al. 2009) will be used to control some confounding variables. Opaque envelopes will be generated and subsequently opened by the operating room anesthesia coordinator prior to surgery that day. The following patients will be excluded:

1. Not wishing to participate in the survey.

2. Absolute contraindications to spinal anesthesia.

3. Difficult airway prediction (possibility of awake intubation.

4. Prior diagnosis of cognitive impairment and / or depression.

5. Chronic use of benzodiazepines (use during last 12 weeks).

6. Referred for postoperative ICU.

7. Anemia (Hemoglobin <10).

8. Current infection.

9. Stage kidney disease> G3a (glomerular filtration rate <45ml / min / 1.73 m2)

10. Body mass index (BMI) characterized by obesity (BMI> 30Kg / m2).

11. History of nausea / vomiting in previous anesthetic procedures.

12. Malignant hyperthermia.

These patients were monitored intraoperatively with cardioscopy, noninvasive pressure, pulse oximetry, capnography, thermometer, bispectral index (BIS) , and neuromuscular blocker monitor.

The patients will be divided into 2 groups:

1) balanced anesthesia + spinal anesthesia with 50 mcg morphine

2) balanced anesthesia + spinal anesthesia with 2.5 ml heavy bupivacaine 0.5% + 50 mcg morphine.

All patients will receive 0.03 mg / kg midazolam intravenous pre-anesthetic medication in the operating room for comfort and 500 ml of crystalloid solution before induction / blockade associated with 4 ml / kg / hour of crystalloid solution plus volume depending on clinical parameters.

Spinal anesthesia will be performed in a sitting position, antisepsis / asepsis with alcoholic chlorhexidine, L3 / L4 space location, 27 G whitacre needle puncture, 0.5 ml shaving and injection lasting 5 to 7 seconds.

Subsequent general anesthesia with pre-oxygenation 8 liters / minute for 5 minutes + fentanyl 3 mcg / kg + propofol 2 mg / kg + rocuronium 0.6 mg / kg will be performed. Anesthesia will be maintained with remifentanil (mcg / kg / min) and desflurane (Fe%).

Hypnosis will be guided by keeping BIS between 40 and 60 and the remifentanil dose will be guided by vital signs.

In both groups, patients will be kept warm with a thermal blanket and their esophageal temperature measured (distance of 40 cm from the incisor teeth) (DANIEI 2008; WANG 2016).

The following parameters will be measured: heart rate, blood pressure, BIS, capnography, saturation, TOF, esophageal temperature and Trendelenburg angle.

The measured moments will be the patient's entry into the operating room, after pre-anesthetic medication, pre-anesthetic induction, after anesthetic induction, after surgical incision, after pneumoperitoneum inflation, after Trendelenburg position, every 15 minutes until the end of the procedure. after extubation.

After the surgical procedure is completed and 5 minutes after extubation, vital signs (blood pressure, heart rate, saturation) will be collected and the patient will be evaluated for delirium. Dipyrone 2 grams and parecoxib 40 mg will be performed with no contraindication for postoperative analgesia.

Afterwards, patients will be referred to the anesthetic recovery room (PACU), where they will continue to be monitored with ECG, noninvasive pressure and pulse oximeters. They will also be evaluated for pain on the numerical rating scale (NRS) (0 = no pain, up to 10 = worst pain), on arrival and every 30 minutes until discharge to the room. Those with NRS> 4 on the PACU will be treated with morphine 1 mg every 10 min or NRS <4. Those with nausea / vomiting will be treated with alizapride 50 mg.

Patients will be assessed for delirium onset using the Confusion Assemenment Method (CAM) (SUPPLEMENT1) in combination with the Richmond Agitation Sedation Scale (RASS) scale (SUPPLEMENT 2).

This evaluation will be performed by nurses, previously trained, upon arrival at the PACU, every 30 minutes until discharge from the post-anesthetic recovery room and every 24 hours until discharge, by the researcher himself. If the patient has an episode of delirium that persists for more than 1 hour, a psychiatrist will be asked for follow-up and treatment.

SUPPLEMENT 1 – Confusion Assement Method - CAM

Confusion Assement Method - CAM

1. Acute onset

There is evidence of acute change in the underlying mental state of the patient? (YES NO)

2. Attention disorder

Did the patient have difficulty focusing his attention, for example, easily distracted or had difficulty keeping up with what was being said? (YES) (NO)

3. Disorganized Thinking

Was the patient's thinking disorganized or inconsistent, with scattered or irrelevant conversion, unclear or illogical flow of ideas, or unpredictable change of subject? (YES) (NO)

4. Change in level of consciousness

Does the patient have altered level of consciousness such as lethargy, numb, comatose? (YES) (NO)

Criteria 1 and 2 plus 3 or 4 must be present.

Adapted from: (INOUYE SK, VAN DICK CH, ALESSI CA 1990)

SUPPLEMENT 2 - Richmond Agitation Sedation Scale – RASS

Richmond Agitation Sedation Scale – RASS

Score	Term	Description
+4	Aggressive	Very aggressive, violent, immediate danger to the team.
+3	Very agitated	Pulls or removes tubes and catheters; aggressive.
+2	Agitated	Frequent unintentional movements, fight against ventilation.
+1	Restless	Anxious, but without aggressive or vigorous movements.
0	Alert and calm	Alert and calm
-1	Sleepy	Not fully alert, but sustained awakening (eye opening / eye contact) at the sound of the voice (> 10 seconds).
-2	Light sedation	Awakens briefly and makes eye contact with voice sound (<10 seconds).
-3	Moderate sedation	Eye movements or opening at the sound of the voice (but without eye contact).
-4	Deep sedation	No response to sound of voice, but moves or opens eyes with physical stimulation.
-5	Not awakened	No response to voice sound or physical stimulation.

Adapted from: (SESSLER et al. 2002)
