# Supplementary material for: Spinal block and delirium in oncologic patients after laparoscopic surgery in the Trendelenburg position: A randomized controlled trial
Source: PLoS One. 2021 May 17;16(5):e0249808. doi: 10.1371/journal.pone.0249808 (PMC8128254; doi:10.1371/journal.pone.0249808)
Supplement: S2 File — (PDF) [file pone.0249808.s002.pdf]

RBR-9yrqwh
Analysis of two anesthetic techniques in the appearance of postoperative delirium of cancer patients submitted to surgeries of videolaparoscopy in trendelenburg position

Registration Date: Oct. 18, 2017, 7:49 a.m.

Last Update: Oct. 29, 2019, 1:10 p.m.

Study Type:

Intervention Study

Scientific Title:

PT-BR	EN
Análise de duas técnicas anestésicas no aparecimento do delirium pós-operatório de pacientes oncológicos submetidos a cirurgias de videolaparoscopia em posição de trendelenburg	Analysis of two anesthetic techniques in the appearance of postoperative delirium of cancer patients submitted to surgeries of videolaparoscopy in trendelenburg position

Trial Identification

UTN Number: U1111-1203-9689

Public Title:

PT-BR	EN
A influência do tipo de anestesia no surgimento de alterações da atividade cerebral após cirurgias realizadas com video, nas quais a cabeça do paciente fica inclinada para baixo	The influence of the type of anesthesia on the appearance of changes in brain activity after video surgeries, in which the patient's head is tilted downwards

Scientific Acronym:

Public Acronym:

Secondary Identifying Numbers:

2.125.810
Issuing Authority: Comitê de Ética em Pesquisa do Hospital A.C. Camargo Cancer Center

55794216.1.0000.5432
Issuing Authority: Plataforma Brasil

Sponsors

Primary Sponsor: Fundação Antônio Prudente

Secondary Sponsors:

Institution: A.C. Camargo Cancer Center

Source(s) of Monetary or Material Support:

Institution: A.C. Camargo Cancer Center

Health Conditions

Health Condition(s) or Problem(s):

PT-BR
Delirium pós-operatório

EN
Postoperative delirium

General Descriptors for Health Condition(s):

PT-BR
C10: Doenças do sistema nervoso

ES
C10: Enfermedades del sistema nervioso

EN
C10: Nervous system diseases

Specific Descriptors for Health Condition(s):

PT-BR
F03.615: Transtornos Neurocognitivos

ES
F03.615: Trastornos Neurocognitivos

EN
F03.615: Neurocognitive Disorders

Interventions

Intervention Code(s)

Procedure/surgery

Interventions:

PT-BR
Os pacientes serão divididos em 2 grupos (65 pacientes cada):
1) Anestesia geral balanceada.
2) Anestesia geral balanceada associada ao bloqueio subaracnóideo com bupivacaína pesada 0,5% (3ml).
Todos os pacientes receberão morfina (50 mcg por via espinhal).
Esses pacientes serão monitorizados no intra-operatório com eletrocardiograma (ECG), pressão não invasiva, oximetria de pulso, capnografia, BIS, TOF, temperatura esofagiana e ângulo do Trendelenburg.
Todos os pacientes receberão em sala operatória a medicação pré-anestésica endovenosa midazolan 0,03 mg/kg para conforto do mesmo durante o bloqueio subaracnóideo, e 500ml de solução cristaloide antes do bloqueio associados a 4 ml/kg/hora de solução cristaloide, mais volume a depender de parâmetros clínicos.
O bloqueio subaracnóideo será realizado na

EN
Patients will be divided into 2 groups (65 patients each):
1) Balanced general anesthesia.
2) Balanced general anesthesia associated with subarachnoid block with 0.5% hyperbaric bupivacaine (3ml).
All patients will receive morphine (50 mcg via the spinal route).
These patients will be monitored intraoperatively with electrocardiogram , noninvasive pressure, pulse oximetry, capnography, BIS, TOF, esophageal temperature and Trendelenburg angle.
All patients will receive intravenous pre-anesthetic medication midazolam 0.03 mg / kg for comfort during subarachnoid block, and 500 ml of pre-blocking crystalloid solution associated with 4 ml / kg / hour of crystalloid solution, plus depending on clinical parameters.
Subarachnoid block will be performed in the seated position, antisepsis / asepsis with

posição sentada, antissepsia/assepsia com clorexidine alcoólico, localização do espaço L3/L4, punção com agulha whitacre 27 G, barbotagem de 0,5 ml e injeção com duração de 5 a 7 segundos.

A morfina por via espinhal será realizada com a mesma técnica anestésica descrita no bloqueio subaracnóideo.

Na sequência, será realizada anestesia geral com pré-oxigenação 8 litros/minuto durante 5 minutos + fentanil 3 mcg/kg + propofol 2 mg/kg + rocurônio 0,6 mg/kg. Os pacientes serão ventilados com uma mistura de oxigênio e ar através de uma fração inspirada (FI) de oxigênio a 40% e mantendo uma concentração expirada de dióxido de carbono (ETCO2) entre 35 e 45 mmHg. A anestesia será mantida com remifentanil (ng/ml) através do modelo farmacocinético de Minto e desflurano (Fe%).

Hipnose será guiada mantendo BIS entre 40 e 60 e a dose de remifentanil será ajustada pelos sinais vitais.

O bloqueio neuromuscular será mantido com nível profundo durante toda a videolaparoscopia com o objetivo de propiciar um melhor campo operatório para o cirurgião. A dose utilizada, quando necessária, será de um terço da dose de rocurônio utilizada na indução.

A monitorização do bloqueio neuromuscular será realizada com o monitor TOF através do músculo orbicular do olho.

Pacientes com pressões arteriais médias com valores menores que 60 mmHg serão medicados com vasopressores a depender da frequência cardíaca: se maior que 60 batimentos por minuto (bpm), será utilizado metaraminol 0,5 mg; e efedrina 5 mg, se menor que 60 bpm.

Como prevenção de náuseas e vômitos, os pacientes receberão dexametasona 4mg no início do procedimento, e ondansetrona 8 mg no final, desde que não tenha contraindicação.

Em ambos os grupos, os pacientes serão mantidos aquecidos com manta térmica e terão sua temperatura esofagiana mensurada (distância de 40 cm dos dentes incisivos).

Finalizado o procedimento cirúrgico, os portais serão infiltrados com ropivacaína 0,75% (máximo de 3 mg/kg) nos casos sem bloqueio subaracnóideo e o bloqueio neuromuscular será antagonizado com suggamadex, conforme a monitorização do bloqueio neuromuscular através do TOF.

• Bloqueio profundo (pelo menos 1 a 2

alcoholic chlorhexidine, location of the L3 / L4 space, needle puncture with whitacre 27 G, barbotation of 0.5 ml and injection lasting 5 to 7 seconds.

Spinal morphine will be performed with the same anesthetic technique described in the subarachnoid block.

Subsequently, general anesthesia with pre-oxygenation 8 liters / minute for 5 minutes + fentanyl 3 mcg / kg + propofol 2 mg / kg + rocuronium 0.6 mg / kg will be performed. Patients will be ventilated with a mixture of oxygen and air through a 40% inspired fraction of oxygen (FI) and maintaining an expired concentration of carbon dioxide (ETCO2) between 35 and 45 mmHg. Anesthesia will be maintained with remifentanil (ng / ml) through Minto's pharmacokinetic model and desflurane (Fe%).

Hypnosis will be guided by maintaining BIS between 40 and 60 and the dose of remifentanil will be adjusted by vital signs.

The neuromuscular block will be maintained at a deep level throughout the laparoscopy with the objective of providing a better operative field for the surgeon. The dose used, when necessary, will be one third of the dose of rocuronium used in induction. Monitoring of neuromuscular blockade will be performed with the TOF monitor through the orbicularis oculi muscle.

Patients with medium arterial pressures with values lower than 60 mmHg will be medicated with vasopressors depending on heart rate: if greater than 60 beats per minute (bpm), metaraminol 0.5 mg will be used; and ephedrine 5 mg, if less than 60 bpm.

As prevention of nausea and vomiting, patients will receive dexamethasone 4mg at the start of the procedure, and ondansetron 8mg at the end, provided no contraindication.

In both groups, the patients will be kept warm with a thermal blanket and will have their esophageal temperature measured (40 cm from the incisor teeth).

After the surgical procedure, portals will be infiltrated with 0.75% ropivacaine (maximum 3 mg / kg) in cases without subarachnoid block, and the neuromuscular block will be antagonized with suggamadex, according to the monitoring of neuromuscular blockade through TOF.

• Deep block (at least 1 to 2 responses in PTC, but prior to the appearance of T2, ie the second response to TOF: 4 mg / kg.

• Moderate block (after onset of T2): 2mg /

respostas no PTC, mas anterior ao aparecimento de T2, ou seja, a segunda resposta ao TOF: 4 mg/kg.
• Bloqueio moderado (após aparecimento de T2): 2mg/kg.
• 16 mg/kg são recomendados se existir uma condição clinica que necessite de reversão rápida (aproximadamente 3 minutos) após a administração de dose única de rocurônio a 1,2 mg/kg.
Após 5 minutos da extubação, os sinais vitais (pressão arterial, frequência cardíaca, saturação) serão coletados e o paciente será avaliado com relação à presença de delirium. Serão aplicados, para analgesia de pós-operatório, dipirona (2 gramas) e parecoxibe (40 mg), não havendo contraindicação.

kg.
• 16 mg / kg are recommended if there is a clinical condition requiring rapid reversal (approximately 3 minutes) following the single dose of rocuronium at 1.2 mg / kg.
After 5 minutes of extubation, vital signs (blood pressure, heart rate, saturation) will be collected and the patient will be evaluated for the presence of delirium. Dipyrone (2 grams) and parecoxib (40 mg) will be used for postoperative analgesia, and there is no contraindication.

Descriptor for Intervention(s):

PT-BR
E03: Anestesia e Analgesia

ES
E03: Anestesia y Analgesia

PT-BR
E03.155.086.131.100: Anestesia Caudal

ES
E03.155.086.131.100: Anestesia Caudal

Recruitment

Recruitment Status: Recruiting

Recruitment Country

Brazil

Planned Date of First Enrollment: 2017-10-18

Planned Date of Last Enrollment: 2018-09-25

Target Sample Size:	Gender (inclusion sex):	Inclusion Minimum Age:	Inclusion Maximum Age:
130	-	18 -	0 -

Inclusion Criteria:

PT-BR
pacientes do hospital AC Camargo Cancer Center; 18 anos ou mais, ASA (classificação American Society of Anesthesia) inferior a 3; submetidos a cirurgia eletiva videolaparoscópica em posição de Trendelenburg, ficando nesta posição por um período mínimo de 2 horas e que concordarem em assinar o termo de consentimento livre e esclarecido.

EN
patients from the AC Camargo Cancer Center; aged 18 years or over; American Society of Anesthesia (ASA), less than 3, submitted to elective laparoscopic surgery in a Trendelenburg position, minimum period of 2 hours and who agree to sign the free and informed consent form.

Exclusion Criteria:

PT-BR	EN
Contraindicações absolutas e relativas ao bloqueio subaracnóideo; com previsão via aérea difícil com possibilidade de intubação acordado); com diagnóstico prévio de: distúrbio cognitivo e/ou depressão; fazendo uso crônico de benzodiazepínicos (uso durante as ultimas 12 semanas) ;encaminhados para realizar o pós-operatório em UTI; com diagnóstico de anemia (Hemoglobina < 10 mg/dl); com diagnóstico de infecção atual; com doença renal com estágio > G3a (taxa de filtração glomerular < 45 ml/min/1.73 m2); com índice de massa corpórea (IMC) caracterizado por obesidade (IMC > 30 Kg/m2); com história de náuseas/vômitos em procedimentos anestésicos prévios;com diagnóstico de hipertermia maligna.	Absolute contraindication and related to spinal anesthesia; with difficult airway prediction with possibility of awake intubation; With previous diagnosis of: cognitive disorder and / or depression; chronic use of benzodiazepines (use during the last 12 weeks); referred to perform the postoperative ICU; with diagnosis of anemia (Hemoglobin <10); with diagnosis of current infection; with renal disease with stage> G3a (glomerular filtration rate <45 ml / min / 1.73 m2; with body mass index (BMI) characterized by obesity (BMI> 30 kg / m2);with history of nausea / vomiting in previous anesthetic procedures; with diagnosis of malignant hyperthermia.

Study Type

Study Design:

PT-BR	EN
Ensaio clínico de prevenção, randomizado-controlado, paralelo, uni-cego, com dois braços	Trial, randomized-controlled, parallel, uni-blind, two-arm clinical trial

Expanded access program	Study Purpose	Intervention Assignment	Number of arms	Masking type	Allocation type	Study Phase
False	Prevention	Parallel	2	Single-blind	Randomized-controlled	N/A

Outcomes

Primary Outcomes:

PT-BR	EN
Incidência de delirium no pós-operatório com anestesia geral + morfina por via espinhal. Delirium avaliado pela escala CAM (Confusion Assemenment Method) e RASS (Richmond Agitation Sedation Scale)	Incidence of postoperative delirium with general anesthesia + spinal morphine. Delirium assessed by CAM scale Confusion Assemenment Method) e RASS (Richmond Agitation Sedation Scale)

PT-BR	EN
Incidência de delirium no pós-operatório com anestesia geral + bloqueio subaracnoideo. Delirium avaliado pela escala CAM (Confusion Assemenment Method) e RASS (Richmond Agitation Sedation Scale)	Incidence of postoperative delirium with general anesthesia + subarachnoid block. Delirium assessed by CAM scale Confusion Assemenment Method) e RASS (Richmond Agitation Sedation Scale)

Secondary Outcomes:

PT-BR	EN
Não são esperados desfechos secundários	Secondary outcomes are not expected

Contacts

Contacts for Public Queries

Full Name: Giane Nakamura

Address: Rua Professor Antônio Prudente, 211

City: São Paulo / Brazil

Zip Code: 01509-010

Telephone: 55-11-21895000

E-mail: gianenakamura@hotmail.com

Affiliation: Fundação Antônio Prudente

Contacts for Scientific Queries

Full Name: Giane Nakamura

Address: Rua Professor Antônio Prudente, 211

City: São Paulo / Brazil

Zip Code: 01509-010

Telephone: 55-11-21895000

E-mail: gianenakamura@hotmail.com

Affiliation: Fundação Antônio Prudente

Contact(s) for Site Queries

Full Name: Giane Nakamura

Address: Rua Professor Antônio Prudente, 211

City: São Paulo / Brazil

Zip Code: 01509-010

Telephone: 55-11-21895000

E-mail: gianenakamura@hotmail.com

Affiliation: Fundação Antônio Prudente

Previous Revision

Additional Links:

Download as ICTRP format

Download as OpenTrials XML format
