# Supplementary material for: Spinal block and delirium in oncologic patients after laparoscopic surgery in the Trendelenburg position: A randomized controlled trial
Source: PLoS One. 2021 May 17;16(5):e0249808. doi: 10.1371/journal.pone.0249808 (PMC8128254; doi:10.1371/journal.pone.0249808)
Supplement: S1 Protocol — (DOCX) [file pone.0249808.s004.docx]

**Spinal block and delirium in oncologic patients after laparoscopic surgery in the Trendelenburg position: A randomized controlled trial**

**JORGE KIYOSHI MITSUNAGA JUNIOR**

**Dissertation presented to Fundação Antônio Prudente to obtain the title of PhD of Science**

**Concentration Area: Oncology**

**Advisor: Dr. Giane Nakamura**

**São Paulo 2017**

1. **INTRODUCTION**

Cancer has had a huge impact on our society for a long time and as a result it has been an important topic in all the discussions.

According to world estimates by the World Health Organization (WHO), 14 million new cancer cases and a total of 8.2 million cancer deaths were detected worldwide in 2012. These figures are alarming and may be become even greater if broad preventive measures are not taken (Ministry of Health 2016).

With regard to Brazil, the estimate for 2016/2017 points to the occurrence of about 600 thousand new cases of cancer. Except for non-melanoma skin cancer (approximately 180 thousand new cases), there will be approximately 420 thousand new cases of cancer (Ministério da Saúde 2016).

The normative framework in the area of ​​oncology in the Unified Health System (SUS) is based on two ordinances and it is considered that for every 1000 new cases of cancer, it is expected that 500 to 600 will require oncological surgery, 700 for chemotherapy and 600 for radiotherapy. In 2010, 44,580 oncology surgeries were performed in the state of São Paulo alone (CECILIOII and TAMELINIII 2011).

These surgeries, which in their vast majority are large, involving large resections, subject to a high degree of pain in the postoperative period and consequently lead to a prolonged period of recovery. With regard to patients, they have several comorbidities and they underwent various procedures and treatments, especially chemotherapy and radiotherapy (VERONESI and STAFYLA 2012).

In the era of minimally invasive procedures, laparoscopic surgery has been gaining unanimity (HENNY and HOFLAND 2005). Among its numerous advantages are: less pain in the postoperative period, better aesthetic results due to small incisions, quick return to daily activities, reduction in hospitalization days, less intraoperative bleeding, less pulmonary complications in the postoperative period, less infection of surgical wound, less metabolic breakdown in the postoperative period and consequently cost reduction (GERGES et al. 2006).

This technique consists of creating the pneumoperitoneum, which is the insufflation of a gas through a small incision in the abdominal wall in order to separate it from Organs internal organs, allowing a better visibility of the area to be treated by the surgeon and manipulation of the instruments within the cavity. (BIRCH DW, DANG JT, SWITZER NJ, MANOUCHEHRI N, SHI X, HADI G 2016).

The pneumoperitoneum pressure is defined as normal and adequate when the values ​​are between 12 to 15 mmHg, being considered low when the values ​​are between 5 to 7 mmHg, below this last band it would be practically impossible to practice this surgical technique, because the visualization of structures would be greatly impaired (NEUDECKER et al. 2002). The increase in this pressure leads to an increase in gas absorption, elevates the diaphragm, decreasing lung compliance and increasing airway pressure. Increased intra-abdominal pressure increases venous return due to compression of the splanchnic vasculature. Pneumoperitoneum also increases systemic vascular resistance and pulmonary vascular resistance. During the initial phase of the pneumoperitoneum, cardiac output is reduced by decreasing venous return. Although these changes are well tolerated by healthy individuals with a good cardiopulmonary reserve, patients without this reserve may not tolerate as well (GURUSAMY KS, VAUGHAN J 2014). Approximately 17% of patients undergoing laparoscopic cholecystectomy are ASA III or IV (GIGER et al. 2006), whereas in oncological surgeries this number may be even higher.

The ideal gas would be one with the following properties: minimum peritoneal absorption, minimum physiological effects, rapid excretion of any absorbed gas, non-oxidizing, minimal effect of intravascular embolization and high blood solubility (GERGES et al. 2006).

Among the available gases, the most commonly used is carbon dioxide because it has characteristics closer to the ideal gas, but it also has undesired characteristics (GERGES et al. 2006). Due to its diffusibility characteristic, the pneumoperitoneal caused by carbon dioxide is dissolved quickly, however despite the speed, a certain amount can remain and cause discomfort. Due to its high solubility it can cause hypercarbia, hypercapnia and acidosis (MENES and SPIVAK 2000).

As soon as pneumoperitoneum is established, there is an increase in intra-abdominal pressure and, consequently, cardiovascular, respiratory and neurological effects (CONACHER et al. 2004; HENNY and HOFLAND 2005; GERGES et al. 2006; KALMAR et al. 2010).

The severity of possible cardiovascular effects such as blood pressure variation, arrhythmias and cardiac arrest are related to the volume of carbon dioxide absorbed, the patient's intravascular volume, ventilatory technique, surgical conditions, anesthetic agents used, however the most important factors are intra pressure -abdominal and position of the patient (HENNY and HOFLAND 2005; GERGES et al. 2006).

Respiratory effects include reduced lung volume, increased airway pressure, decreased pulmonary compliance secondary to increased intra-abdominal pressure and patient position (CONACHER et al. 2004; HENNY and HOFLAND 2005; GERGES et al. 2006; KALMAR et al. 2010).

Neurological changes arise due to increased intracranial pressure, decreased cerebral perfusion, hypercapnia and increased systemic vascular resistance (CONACHER et al. 2004; GERGES et al. 2006; KALMAR et al. 2010).

Regarding the positioning of the patient for laparoscopic surgeries, the most diverse are described, but one of the most challenging and with the most physiological changes occurs in those that need to be performed in the Trendelenburg position (MARTIN JT 1997).

Headache or better known as Trendelenburg position was described in the middle of the 19th century, by a German surgeon Friedrich Trendelenburg, and continues to be used routinely today, mainly in surgeries of the genitourinary or colorectal tracts. One of the great advantages of using this position is the improvement of the exposure of the surgical field providing the improvement of the surgical technique (MARTIN 1997; TALAB et al. 2016). Patients with prolonged duration in this position may have edema of the face, conjunctiva, larynx and tongue with a potential risk of airway obstruction. Special care must also be taken with the patient's positioning, as he becomes subject to a nervous plexus injury (ANDREA CESTARI and MARIA BUFFI, EMANUELE SCAPATICCI, GIOVANNI LUGHEZZANI, ANDREA SALONIA, ALBERTO BRIGANTI, PATRIZIO RIGATTI, FRANCESCO MONTORSI) (MICHEL W. COPPIETERS 2002).

Some disadvantages of this procedure associated with the Trendelenburg position would be pulmonary pathophysiological changes with the formation of atelectasis and increased airway pressure. They are also associated with increased intracranial pressure and the formation of cerebral edema. These effects could lead to low cerebral perfusion and potential mismatch of oxygen supply to the brain (CONACHER et al. 2004; CLOSHEN et al. 2014; ROSENDAL et al. 2014).

For laparoscopic surgeries, general anesthesia with short-term agents is almost unanimous. With regard to the technique for maintaining general anesthesia, there are currently two techniques: total intravenous anesthesia and inhalation anesthesia. Both are universally used, with rare exceptions for one technique to be more advantageous compared to the other technique, as in certain specific situations. Total intravenous anesthesia would be better indicated for patients with a history of nausea / vomiting in the postoperative period (APFEL et al. 2002; LEE et al. 2015), it is not a triggering agent of malignant hyperthermia (HOPKINS 2000; MANI and MORTON 2010) less disorders of behavior in the postoperative period of pediatric surgery (ORTIZ et al. 2014), anesthesia in neurosurgery with a tendency to intracranial hypertension (PETERSEN et al. 2003). Inhalation anesthesia would be more beneficial with neuroprotection (KITANO et al. 2007), cardioprotection (FRABDORF et al. 2009; PS 2010) and less chance of intraoperative recall (AMERICAN SOCIETY OF ANESTHESIOLOGISTS TASK FORCE ON INTRAOPERATIVE AWARENESS 2006).

The association with spinal anesthesia, provided that the guidelines for its contraindications are followed (TABLE 1), is also seen as advantageous, since it produces earlier awakening, reduces nausea and vomiting, postoperative pain, length of hospital stay, effective cost, improvement patient satisfaction (BESSA et al. 2012; CARD et al. 2015; DAY et al. 2015; GERGES et al. 2006; GHOSH et al. 2015; SINHA et al. 2009; WONGYINGSINN et al. 2012) and mainly the decrease consumption of anesthetic agents for general anesthesia (GERGES et al. 2006).

Chart 1 - Contraindications to spinal anesthesia.

| Absolute | Relative |
| --- | --- |
| Patient refusal | Coagulopathy |
| Significant hypovolemia | Sepsis / bacteremia |
| Infection at the puncture site | Previous neurological disease |
| Intracranial hypertension | Peripheral neuropathy |
| Significant coagulopathy | Multiple sclerosis |
|  | Demyelinating process |

As a disadvantage, when associating the two anesthetic techniques, patients become susceptible to the adverse events inherent to spinal anesthesia. Among these, the most common are hypotension of around 5% (CARPENTER, RL; CAPLAN, RA; BROWN 1992; HARTMANN et al. 2002),

nausea and vomiting described in 15% (BORGEAT et al. 2003), pruritus varies from 30 to 100% of patients (RATHMELL et al. 2005), urinary retention was observed in up to 35% of patients (KUIPERS et al. 2004), tremors, which can occur in up to 50% of patients (CROWLEY and BUGGY 2008) (CROWLEY and BUGGY 2008). Intrinsic complications to the procedure are rare, but described in the literature, such as hematoma, infection, and fatal cardiovascular glue in 1 / 100,000 patients (COOK et al. 2009; MOEN et al. 2004).

Another possibility would be spinal anesthesia with opioids alone, more commonly morphine (ARAIMO MORSELLI et al. 2016; DICHTWALD et al. 2016), bringing the benefit of analgesia for a period of 12 to 24 hours, without the cardiovascular consequences caused by the blockade sympathetic as a consequence of using the local anesthetic (ARAIMO MORSELLI et al. 2016).

Currently, in laparoscopic surgeries, it is not clear which is the best anesthetic technique for such a procedure. There are attempts to search for indications of spinal anesthesia through systematic literature reviews (PROSPECT [s.d.]) or guidelines (AMERICAN SOCIETY OF ANESTHESIOLOGISTS TASK FORCE ON ACUTE PAIN MANAGEMENT 2012), however none is categorical and with a strong level of evidence in affirming such conduct.

In the postoperative period, one of the most feared neurological changes is delirium, being considered the most common post-surgical complication, occurring between 5 to 50% of the procedures (INOUYE et al. 2014). The same is a perverse and complicated process that brings countless challenges to the team that accompanies the patient (ALDECOA et al. 2017; STEINER 2011a). Especially harmful in the elderly, since a single episode can trigger a cascade of events such as: prolonged hospitalization, loss of functional independence, reduction of cognitive function and death (SACZYNSKI JS, MARCANTONIO ER, QUACH L, FONG TG, GROSS A, INOUYE SK 2012). Some meta-analyzes suggest that a single episode of delirium increases the risk of death by 2 times, increases the time of mechanical ventilation, time in the ICU and hospital stay (MCDANIEL and BRUNEY 2012).

Delirium is defined in the DSM V (Diagnostic and Statiscal Manual of Mental Disorders) as: disturbance of consciousness, alteration of cognition and acute and fluctuating course time (AMERICAN PSYCHIATRIC ASSOCIATION 2013).

Clinically, there are several methods for diagnosing delirium, but the most widely used is performed using the Confusion Assemenment Method (CAM) (FABBRI et al. 2001; STEINER 2011b) (ANNEX 1), a simple screening method based on four questions that it has a sensitivity of 86% and a specificity of 93% (WONG et al. 2010). The checklist for diagnosing delirium consists of 4 items: 1 - Acute onset; 2- Attention disorder; 3- Disorganized thinking; 4 - Change in the level of consciousness. The diagnosis of delirium requires the presence of criteria 1 and 2 plus criteria 3 or 4.

A variant known as CAM-ICU is often used in intubated or sedated patients (ELY et al. 2001; LUETZ et al. 2010), but at present there is still no specific and well-established method for evaluating the delirium of surgical patients in immediate postoperative period.

There are 3 subtypes of delirium: hyperactive (agitation and restlessness) (25%), hypoactive (lethargy and inattention) (50%) and mixed (25%). In a study involving 400 patients, with a median age of 57 (44.67) years, evaluated in the anesthetic recovery room, 124 (31%) had delirium on admission to the anesthetic recovery room, 59 (15%) 30 minutes after admission, 32 (8%) with 60 minutes after admission and 15 (4%) at discharge from the anesthetic recovery room. In patients with signs of delirium, hypoactive signs were present in 56% on admission to the anesthetic recovery room and in 92% during their stay in the anesthetic recovery room (CARD et al. 2015). The hypoactive subtype is the most prevalent and also associated with higher mortality. Although there is no consensus on the definition of postoperative delirium, most studies define its occurrence between 24 to 72 hours postoperatively, however some still occur after discharge from hospital (WHITLOCK et al. 2011; CARD et al. 2015).

The Richmond Agitation Sedation Scale (RASS) scale, developed with the objective of characterizing the level of consciousness and agitation, is routinely used in association with CAM to characterize delirium. Signs of hyperactive delirium are defined with a RASS score ranging from +1 (anxious patient) to +4 (agitated patient) accompanied by positive CAM. Signs of hypoactive delirium will be defined as RASS ranging from - 5 (patient does not respond) to 0 (calm or sleepy patient) accompanied by negative CAM (NASSAR et al. 2008; SESSLER et al. 2002) (ANNEX 2).

Currently, the most accepted hypothesis for the pathophysiology of delirium in the postoperative period seems to be an acute neurological insult in a patient with a susceptible substrate. Such a disjunction results from an alteration in the balance of neurotransmitters or neuroinflammatory mediators in the brain, resulting in clinical symptoms of delirium. The most commonly affected pathways include acetylcholine, dopamine, gamma aminobutyric acid (GABA) and serotonin, where the most common neuroinflammatory marker involves the hypothalamic pituitary axis, represented by cortisol, as well as C-reactive protein (CRP), pro calcitonin, tumor necrosis and various interleukins such as IL-6, IL-8 and IL-10 (ALI et al. 2011). Although there is general acceptance of which clinical indicators define the susceptible substrate, there has been an increase in efforts to identify preoperative and postoperative biochemical markers that may help to identify potential risks for delirium based on the aforementioned theories (MCDANIEL and BRUNEY 2012) .

The assumed risk factors are multifactorial and these can be grouped into non-modifiable and modifiable. In the first group there is an increase in age, a poor functional status already detected in the preoperative period, comorbidities (dementia, depression, nephropathy, heart disease and lung diseases), type of surgery (emergency, orthopedics, cardiovascular) (SCHOEN et al. 2011 ). The second group includes certain classes of drugs (opioid, benzodiazepine, antihistamine, dihydropyridine) (CLEGG and YOUNG 2011), polypharmacy (more than six drugs or the addition of three new ones), infection or inflammation, pain, electrolyte disturbance ( sodium and potassium), hematological changes (anemia, hypoxemia), changes in the sleep-wake cycle (WHITLOCK et al. 2011; SANDERS et al. 2011; CHAPUT and BRYSON 2012), pressure of intraoperative erfusion and possibly depth of the anesthetic plan (SIEBER et al. 2010).

Regarding the use of drugs, opioids should be prescribed with caution, since their use, especially in high doses, could increase the risk of delirium. On the other hand, severe pain not properly treated is associated with its appearance. Regarding benzodiazepines, the association occurs mainly with high doses and with long-lasting drugs (CLEGG and YOUNG 2011).

It is common knowledge that a preoperative assessment and identification of risk factors greatly reduces the chances of the event (ADULTS 2014).

One of the ways found to try to reduce the incidence of delirium during the operation is through the modernization of monitoring. Regarding multimodal monitoring (cardiac output, depth of hypnosis, cerebral oxygenation), this has been gaining a lot of focus. By detecting and reducing complications, it contributes to videolaparoscopy to come closer to the ideal surgery method (GREEN et al. 2014).

Neuromonitoring during anesthetic procedure can improve the patient's awakening (GUARRACINO 2008; FEDOROW and GROCOTT 2010). The BIS (Bi-Spectral Index) is derived from an analysis of the electroencephalogram (EEG) and represents the anesthetic depth through a single value that can vary from 0 to 100 and that correlates with sedation and hypnosis, being used clinically for anesthetic agents. Values ​​between 40 and 60 are said to be suitable for surgery (AG et al. 2016). It was one of the first EEG indices and became one of the most used monitors for this purpose in the world. Through it, it is possible to perform hypnosis at the appropriate levels to perform the procedure (PUNJASAWADWONG et al. 2010; KLOPMAN and SEBEL 2011). BIS-guided hypnosis could influence cognitive level and postoperative mortality (FARAG et al. 2006; MONK et al. 2005), however it is still controversial (KERTAI et al. 2011; LESLIE and SHORT 2011).

Today much is said about Triple Low, where patients with low Bispectral Index Scores (BIS) with low concentration of anesthetic agents associated with low perfusion pressure have high rates of morbidity and mortality in 30 to 90 days (DEINERSTACIEANDJEFFREYH.2013).

**2 JUSTIFICATION**

Internationally, the number of laparoscopic surgeries has been gradually increasing (DANIEL J. RISKIN, MICHAEL T. LONGAKER, MICHAEL GERTNER 2006; KAVIC 1998) and among the anesthetic techniques available, general anesthesia and general anesthesia associated with spinal anesthesia, are among the most used (GERGES et al. 2006; HENNY and HOFLAND 2005).

Due to the physiological changes caused by the pneumoperitoneum (HENNY and HOFLAND 2005; KALMAR et al. 2010) associated with the Trendelenburg position (CLOSHEN et al. 2014; KALMAR et al. 2010), it is speculated the increase in the incidence of delirium in the post -operative of these surgeries.

Thus, due to the lack of published studies on the incidence of delirium, in the postoperative period in laparoscopic oncological surgeries in the Trendelenburg position, it was decided to perform the same.

**3 OBJECTIVES**

The aim of this study was to analyze whether the anesthetic techniques used for laparoscopic oncological surgeries in the Trendelenburg position, differed in relation to the incidence of postoperative delirium.

**MATERIAL AND METHODS**

Prospective, randomized study , including patients from the operating room of AC Camargo Hospital, aged 18 years or older, ASA less than 3, undergoing laparoscopic elective surgery in a Trendelenburg position lasting at least 2 hours in the cephalodeclive and agree to sign the free and informed consent form.

Study will be evaluated by the ethics and research committee (CEP) of the Antônio Prudente Foundation (FAP).

Patients will be sequentially allocated to possible 2 groups. Sequential allocation (FOSSALUZA et al. 2009) will be used to control some confounding variables. Opaque envelopes will be generated and subsequently opened by the operating room anesthesia coordinator prior to surgery that day. The following patients will be excluded:

1. Not wishing to participate in the survey.
2. Absolute contraindications to spinal anesthesia.
3. Difficult airway prediction (possibility of awake intubation.
4. Prior diagnosis of cognitive impairment and / or depression.
5. Chronic use of benzodiazepines (use during last 12 weeks).
6. Referred for postoperative ICU.
7. Anemia (Hemoglobin <10).
8. Current infection.
9. Stage kidney disease> G3a (glomerular filtration rate <45ml / min / 1.73 m2)

10. Body mass index (BMI) characterized by obesity (BMI> 30Kg / m2).
11. History of nausea / vomiting in previous anesthetic procedures.
12. Malignant hyperthermia.

These patients were monitored intraoperatively with cardioscopy, noninvasive pressure, pulse oximetry, capnography, thermometer, bispectral index (BIS) , and neuromuscular blocker monitor.

The patients will be divided into 2 groups: 1) balanced anesthesia + spinal anesthesia with 50 mcg morphine

2) balanced anesthesia + spinal anesthesia with 2.5 ml heavy bupivacaine 0.5% + 50 mcg morphine.

All patients will receive 0.03 mg / kg midazolam intravenous pre-anesthetic medication in the operating room for comfort and 500 ml of crystalloid solution before induction / blockade associated with 4 ml / kg / hour of crystalloid solution plus volume depending on clinical parameters.

Spinal anesthesia will be performed in a sitting position, antisepsis / asepsis with alcoholic chlorhexidine, L3 / L4 space location, 27 G whitacre needle puncture, 0.5 ml shaving and injection lasting 5 to 7 seconds.

Subsequent general anesthesia with pre-oxygenation 8 liters / minute for 5 minutes + fentanyl 3 mcg / kg + propofol 2 mg / kg + rocuronium 0.6 mg / kg will be performed. Anesthesia will be maintained with remifentanil (mcg / kg / min) and desflurane (Fe%).

Hypnosis will be guided by keeping BIS between 40 and 60 and the remifentanil dose will be guided by vital signs.

In both groups, patients will be kept warm with a thermal blanket and their esophageal temperature measured (distance of 40 cm from the incisor teeth) (DANIEI 2008; WANG 2016).

The following parameters will be measured: heart rate, blood pressure, BIS, capnography, saturation, TOF, esophageal temperature and Trendelenburg angle.

The measured moments will be the patient's entry into the operating room, after pre- anesthetic medication, pre-anesthetic induction, after anesthetic induction, after surgical incision, after pneumoperitoneum inflation, after Trendelenburg position, every 15 minutes until the end of the procedure. after extubation.

After the surgical procedure is completed and 5 minutes after extubation, vital signs (blood pressure, heart rate, saturation) will be collected and the patient will be evaluated for delirium. Dipyrone 2 grams and parecoxib 40 mg will be performed with no contraindication for postoperative analgesia.

Afterwards, patients will be referred to the anesthetic recovery room (PACU), where they will continue to be monitored with ECG, noninvasive pressure and pulse oximeters. They will also be evaluated for pain on the numerical rating scale (NRS) (0 = no pain, up to 10 = worst pain), on arrival and every 30 minutes until discharge to the room. Those with NRS> 4 on the PACU will be treated with morphine 1 mg every 10 min or NRS <4. Those with nausea / vomiting will be treated with alizapride 50 mg.

Patients will be assessed for delirium onset using the Confusion Assemenment Method (CAM) (SUPPLEMENT1) in combination with the Richmond Agitation Sedation Scale (RASS) scale (SUPPLEMENT 2).

This evaluation will be performed by nurses, previously trained, upon arrival at the PACU, every 30 minutes until discharge from the post-anesthetic recovery room and every 24 hours until discharge, by the researcher himself. If the patient has an episode of delirium that persists for more than 1 hour, a psychiatrist will be asked for follow-up and treatment.

With regard to statistical analysis, due to the lack of studies involving this specific group of patients, a simulation was carried out with the incidence of delirium seen by Sanders et al 2011.

- Proportion of cases among those exposed: 5%
- Proportion of cases among those not exposed: 21%
- Calculated relative risk: 0.2381
- Significance level: 5%
- Test power: 85%
- Hypothesis testing: single-tailed
- Sample size calculated for each group: 62

Considering losses, the number of patients was stipulated for each group (ANNEX 3).

• Group 1 - “n: 65” - balanced anesthesia + spinal anesthesia with + morphine

50 mcg.

• Group 2 - “n: 65” - balanced anesthesia + spinal anesthesia with 2.5 ml

0.5% heavy bupivacaine + 50 mcg morphine.

Initially, a descriptive analysis of the data will be performed, in which the distribution of absolute / relative frequency will be presented for the qualitative variables and the main summary measures, such as mean, median, maximum, minimum and standard deviation will be presented for the quantitative variables. In order to assess a possible association between the variables of interest and the outcome (delirium and non-delirium), the independence test (chi-square or Fisher's exact) will be applied. In addition, in order to assess and quantify the impact of each independent variable on the outcome of interest, the simple and multiple logistic regression model will be adjusted to the data. In all tests, a significance level of 5% will be set. The analyzes will be performed using SPSS software version 23 and free software R version 3.4

**5 RESULTS**

Project in progress.

**6 DISCUSSION**

Project in progress.

**7 CONCLUSION**

Project in progress.

**8 REFERENCES**

Adults TAGSEP ON PD IN O. Postoperative Delirium in Older Adults: Best Practice Statement from the American Geriatrics Society. **Journal of the American College of Surgeons** 2014;

Ag M, Wang M, Mj W, et al. Anaesthetic interventions for prevention of awareness during surgery ( Review ) SUMMARY OF FINDINGS FOR THE MAIN COMPARISON. **Cochrane Library** 2016;

Aldecoa C, Bettelli G, Bilotta F, et al. European Society of Anaesthesiology evidence-based and consensus-based guidelines on postoperative delirium. **European Journal of Anaesthesiology** 2017; 34:192–214.

Ali S, Patel M, Jabeen S, et al. Insight into delirium. **Innovations in Clinical Neuroscience** 2011; 8:25–34.

American Psychiatric Association. **Diagnostic and Statistical Manual of Mental Disorders (DSM-V)**. [s.l: s.n.].

American Society of Anesthesiologists Task Force on Acute Pain Management. Practice guidelines for acute pain management in the perioperative setting: an updated report by the American Society of Anesthesiologists Task Force on Acute Pain Management. **Anesthesiology** 2012; 116:248–73.

American Society of Anesthesiologists Task Force on Intraoperative Awareness. Practice Advisory for Intraoperative Awareness and Brain Function Monitoring. **Anesthesiology** 2006; 104:847–864.

Andrea Cestari N, Maria Buffi, Emanuele Scapaticci, Giovanni Lughezzani, Andrea Salonia, Alberto Briganti, Patrizio Rigatti, Francesco Montorsi GG. Simplifying Patient Positioning and Port Placement During Robotic-Assisted Laparoscopic Prostatectomy. **EUROPEAN UROLOGY** 2010; 57:530–533.

Apfel CC, Kranke P, Katz MH, et al. Volatile anaesthetics may be the main cause of early but not delayed postoperative vomiting: A randomized controlled

trial of factorial design. **British Journal of Anaesthesia** 2002; 88:659–668.

Apfelbaum JL, Hagberg CA CR. Practice guidelines for management of the difficult airway:an updated report by theAmerican Society of Anesthesiologists Task Force on Management of the Difficult Airway. **Anesthesiology** 2013; 118:251–270.

Araimo Morselli FSM, Zuccarini F, Caporlingua F, et al. Intrathecal Versus Intravenous Morphine in Minimally Invasive Posterior Lumbar Fusion: A Blinded Randomized Comparative Prospective Study. **Spine** 2016; 42:281– 284.

Bessa SS, Katri KM, Abdel-Salam WN, El-Kayal E-SA, Tawfik TA. Spinal Versus General Anesthesia for Day-Case Laparoscopic Cholecystectomy: A Prospective Randomized Study. **Journal of Laparoendoscopic & Advanced Surgical Techniques** 2012; 22:550–555.

Birch DW, Dang JT, Switzer NJ, Manouchehri N, Shi X, Hadi G KS. Heated insufflation with or without humidification for laparoscopic abdominal surgery ( Review ). **Cochrane Library** 2016;

Borgeat A, Ekatodramis G, Schenker C. Postoperative nausea and vomiting in regional anesthesia: a review. **Anesthesiology** 2003; 530–547.

Card E, Pandharipande P, Tomes C, et al. Emergence from general anaesthesia and evolution of delirium signs in the post-anaesthesia care unit. **British Journal of Anaesthesia** 2015; 115:411–417.

Carpenter, RL; Caplan , RA;Brown D. Incidence and risk factors for side effects of spinal anesthesia. **Anesthesiology** 1992; 76:906–916.

CecilioII MCMMACMNFMAM, TameliniII RM. Diretrizes para a atenção oncológica no Estado de São Paulo: contribuições para o debate. **BEPA** 2011; 8:24–43.

Chaput AJ, Bryson GL. Postoperative delirium: risk factors and management: continuing professional development. **Canadian journal of anaesthesia = Journal canadien d’anesthésie** 2012; 59:304–20.

Clegg A, Young JB. Which medications to avoid in people at risk of delirium: A systematic review. **Age and Ageing** 2011; 40:23–29.

Closhen D, Treiber A-H, Berres M, et al. Robotic assisted prostatic surgery in the Trendelenburg position does not impair cerebral oxygenation measured using two different monitors: A clinical observational study. **European journal of anaesthesiology** 2014; 31:104–9.

Conacher ID, Soomro NA, Rix D. Anaesthesia for laparoscopic urological surgery. **British Journal of Anaesthesia** 2004; 93:859–864.

Cook TM, Counsell D, Wildsmith JAW. Major complications of central neuraxial block: Report on the Third National Audit Project of the Royal College of Anaesthetists. **British Journal of Anaesthesia** 2009; 102:179–190.

Crowley LJ, Buggy DJ. Shivering and Neuraxial Anesthesia. **Regional Anesthesia and Pain Medicine** 2008; 33:241–252.

DanieI S. Temperature Monitoring and Perioperative Thermoregulation. **Anesthesiology** 2008; 109:318–38.

Daniel J. Riskin, Michael T. Longaker, Michael Gertner TMK. Innovation in Surgery. **Annals of Surgery** 2006; 244:686–693.

Day AR, Smith RVP, Scott MJP, Fawcett WJ, Rockall TA. Randomized clinical trial investigating the stress response from two different methods of analgesia after laparoscopic colorectal surgery. **British Journal of Surgery** 2015; 102:1473–1479.

Deiner Stacie and Jeffrey H. Long-Term Outcomes in Elderly Surgical Patients. **Mt Sinai J of Medicine of Medicine** 2013; 79:95–106.

Dichtwald S, Ben-Haim M, Papismedov L, Hazan S, Cattan A, Matot I. Intrathecal morphine versus intravenous opioid administration to impact postoperative analgesia in hepato-pancreatic surgery: a randomized controlled trial. **Journal of Anesthesia** 2016; 1–9.

Ely EW, Ely EW, Inouye SK, et al. Delirium in Mechanically Ventilated Patients. **Jama** 2001; 286:2703–2710.

Erman AB, Collar RM, Griffith KA, et al. Sentinel lymph node biopsy is accurate and prognostic in head and neck melanoma. **Cancer** 2012; 118:1040–1047.

Fabbri RMA, Moreira MA, Garrido R, Almeida OP. Validity and reliability of the portuguese version of the confusion assessment method (CAM) for the detection of delirium in the elderly. **Arquivos de Neuro-Psiquiatria** 2001; 59:175–179.

Farag E, Chelune GJ, Schubert A, Mascha EJ. Is depth of anesthesia, as assessed by the Bispectral Index, related to postoperative cognitive dysfunction and recovery? **Anesthesia and Analgesia** 2006; 103:633–640.

Fedorow C, Grocott HP. Cerebral monitoring to optimize outcomes after cardiac surgery. **Current opinion in anaesthesiology** 2010; 23:89–94.

Fossaluza V, Diniz JB, De Bragança Pereira B, Miguel EC, De Bragança Pereira CA. Sequential Allocation to Balance Prognostic Factors in a Psychiatric Clinical Trial. **Clinics (Sao Paulo, Brazil)** 2009; 64:511–518.

Foundation NK. **CLINICAL PRACTICE GUIDELINES For Chronic Kidney Disease: Evaluation, Classification and Stratification**. [s.l: s.n.].

Frabdorf J, De Hert S, Schlack W. Anaesthesia and myocardial ischaemia/reperfusion injury. **British Journal of Anaesthesia** 2009; 103:89– 98.

Gerges FJ, Kanazi GE, Jabbour-Khoury SI. Anesthesia for laparoscopy: A review. **Journal of Clinical Anesthesia** 2006; 18:67–78.

Ghosh S, Saha S, Mallik S, Pal S, Das W, Bhattacharya S. Comparison between general anesthesia and spinal anesthesia in attenuation of stress response in laparoscopic cholecystectomy: A randomized prospective trial. **Saudi Journal of Anaesthesia** 2015; 9:184.

Giger UF, Michel J, Opitz I, Inderbitzin DT. Risk Factors for Perioperative Complications in Patients Undergoing Laparoscopic Cholecystectomy : Analysis of 22 , 953 Consecutive Cases from the Swiss Association of Laparoscopic and Thoracoscopic Surgery Database. **Journal of American**

**College of Surgeons** 2006; 723–728.

Green D, Bidd H, Rashid H. Multimodal intraoperative monitoring: An observational case series in high risk patients undergoing major peripheral vascular surgery. **Int J Surg** 2014; 12:231–236.

Guarracino F. Cerebral monitoring during cardiovascular surgery. **Current opinion in anaesthesiology** 2008; 21:50–54.

Gurusamy KS, Vaughan J DB. Low pressure versus standard pressure pneumoperitoneum in laparoscopic cholecystectomy ( Review ). **Cochrane Library** 2014;

Hartmann B, Junger A, Klasen J, et al. The Incidence and Risk Factors for Hypotension After Spinal Anesthesia Induction: An Analysis with Automated Data Collection. **Anesthesia and analgesia** 2002; 94:1521–1529.

Henny CP, Hofland J. Laparoscopic surgery: Pitfalls due to anesthesia, positioning, and pneumoperitoneum. **Surgical Endoscopy and Other Interventional Techniques** 2005; 19:1163–1171.

Hopkins PM. Malignant hyperthermia: advances in clinical management and diagnosis. **British journal of anaesthesia** 2000; 85:118–128.

Inouye SK, Westendorp RGJ, Saczynski JS. Delirium in elderly people. **The Lancet** 2014; 383:911–922.

Inouye SK, Van Dick CH, Alessi CA BS. Clarifying confusion: the confusion assessment method: a new method for detection of deliruium. **Ann Intern Med** 1990; 133:941–8.

Kalmar AF, Foubert L, Hendrickx JFA, et al. Influence of steep Trendelenburg position and CO2 pneumoperitoneum on cardiovascular, cerebrovascular, and respiratory homeostasis during robotic prostatectomy. **British Journal of Anaesthesia** 2010; 104:433–439.

Kavic MS. A decade of laparoscopic cholecystectomy. **JSLS : Journal of the Society of Laparoendoscopic Surgeons / Society of Laparoendoscopic Surgeons** 1998; 2:319–20.

Kertai MD, Palanca BJ A, Pal N, et al. Bispectral index monitoring, duration of bispectral index below 45, patient risk factors, and intermediate-term mortality after noncardiac surgery in the B-Unaware Trial. **Anesthesiology** 2011; 114:545–556.

Kitano H, Kirsch JR, Hurn PD, Murphy SJ. Inhalational anesthetics as neuroprotectants or chemical preconditioning agents in ischemic brain. **Journal of cerebral blood flow and metabolism : official journal of the International Society of Cerebral Blood Flow and Metabolism** 2007; 27:1108–28.

Klopman MA, Sebel PS. Cost-effectiveness of bispectral index monitoring. **Curr Opin Anaesthesiol** 2011; 24:177–181.

Kuipers PW, Kamphuis ET, Van Venrooij GE, et al. Intrathecal opioids and lower urinary tract function: a urodynamic evaluation. **Anesthesiology** 2004; 100:1497–1503.

Lee WK, Kim MS, Kang SW, Kim S, Lee JR. Type of anaesthesia and patient quality of recovery: A randomized trial comparing propofol-remifentanil total i.v. anaesthesia with desflurane anaesthesia. **British Journal of Anaesthesia** 2015; 114:663–668.

Leslie K, Short TG. Low bispectral index values and death: The unresolved causality dilemma. **Anesthesia and Analgesia** 2011; 113:660–663.

Luetz A, Heymann A, Radtke FM, et al. Different assessment tools for intensive care unit delirium: which score to use? **Critical care medicine** 2010; 38:409– 418.

Mani V, Morton NS. Overview of total intravenous anesthesia in children. **Paediatric Anaesthesia** 2010; 20:211–222.

Martin JT WM. **Positioning in Anesthesia and Surgery**. Philadelphia: WB Saunders, 1997.

McDaniel M, Bruney C. Postoperative delirium: etiology and management. **Curr Opin Crit Care** 2012; 18:372–6.

Menes T, Spivak H. Laparoscopy Searching for the proper insufflation gas.

**Surgical Endoscopy** 2000; 1050–1056.

MICHEL W. COPPIETERS MVDVAKHS. Positioning in Anesthesiology: Toward a Better Understanding of Stretch-Induced Perioperative Neuropathies. **Anesthesiology** 2002;

Ministério da Saúde. Estimativa/2016 incidência de câncer no Brasil. **Ministério da Saúde** 2016;

Moen V, Dahlgren N, Irestedt L. Severe Neurological Complications after Central Neuraxial Blockades in Sweden 1990 –1999. **Anesthesiology** 2004; 101:950–9.

Monk TG, Saini V, Weldon BC, Sigl JC. Anesthetic management and one-year mortality after noncardiac surgery. **Anesthesia and Analgesia** 2005; 100:4– 10.

Nassar AP, Neto RCP, De Figueiredo WB, Park M. Validity, reliability and applicability of Portuguese versions of sedation-agitation scales among critically ill patients. **Sao Paulo Medical Journal** 2008; 126:215–219.

Neal JM, Barrington MJ, Brull R, et al. The Second ASRA Practice Advisory on Neurologic Complications Associated With Regional Anesthesia and Pain Medicine Executive Summary 2015. 2015; 40:401–430.

Neudecker J, Sauerland S, Neugebauer E, et al. The European Association for Endoscopic Surgery clinical practice guideline on the pneumoperitoneum for laparoscopic surgery. **Surgical Endoscopy and Other Interventional Techniques** 2002; 16:1121–1143.

Neurologia AB DE PAB DE. Abuso e Dependência de Benzodiazepínicos. **Diretrizes AMB** 2016;

Ortiz AC, Atallah AN, Matos D, Da Silva EM. Intravenous versus inhalational anaesthesia for paediatric outpatient surgery. **The Cochrane database of systematic reviews** 2014; 2:CD009015.

Petersen KD, Landsfeldt U, Cold GE, et al. Intracranial pressure and cerebral hemodynamic in patients with cerebral tumors: A randomized prospective study

of patients subjected to craniotomy in propofol-fentanyl, isoflurane-fentanyl, or sevoflurane-fentanyl anesthesia. **Anesthesiology** 2003; 98:329–336.

Prospect. **Procedure Specific Postoperative Pain Management**. Disponível em: <http://www.postoppain.org>. Acesso em: 15 abr. 2017.

PS P. Cardioprotection by noble gases. **J Cardiothorac Vasc Anesth** 2010; 24:143–163.

Punjasawadwong Y, Phongchiewboon A, Bunchungmongkol N. Bispectral index for improving anaesthetic delivery and postoperative recovery ( Review ) Bispectral index for improving anaesthetic delivery and postoperative recovery. **Cochrane Library** 2010; 10–12.

Rathmell JP, Lair TR, Nauman B. The role of intrathecal drugs in the treatment of acute pain. **Anesthesia and analgesia** 2005; 101:S30–S43.

Rosendal C, Markin S, Hien MD, Motsch J, Roggenbach J. Cardiac and hemodynamic consequences during capnoperitoneum and steep Trendelenburg positioning: Lessons learned from robot-assisted laparoscopic prostatectomy. **Journal of Clinical Anesthesia** 2014; 26:383–389.

Saczynski JS, Marcantonio ER, Quach L, Fong TG, Gross A, Inouye SK JR. Cognitive Trajectories after Postoperative Delirium. **The New England journal of M edicine** 2012; 5:30–39.

Sanders RD, Pandharipande PP, Davidson AJ, Ma D, Maze M. Anticipating and managing postoperative delirium and cognitive decline in adults. **BMJ (Clinical research ed.)** 2011; 343:d4331.

Schoen J, Meyerrose J, Paarmann H, Heringlake M, Hueppe M, Berger K-U. Preoperative regional cerebral oxygen saturation is a predictor of postoperative delirium in on-pump cardiac surgery patients: a prospective observational trial. **Critical Care** 2011; 15:R218.

Sessler CN, Gosnell MS, Grap MJ, et al. The Richmond Agitation – Sedation Scale Validity and Reliability in Adult Intensive Care Unit Patients. **Am J Respir Crit Care Med** 2002;

Sieber FE, Zakriya KJ, Gottschalk A, et al. Sedation depth during spinal anesthesia and the development of postoperative delirium in elderly patients undergoing hip fracture repair. **Mayo Clinic proceedings. Mayo Clinic** 2010; 85:18–26.

Sinha R, Gurwara AK, Gupta SC. Laparoscopic Cholecystectomy Under Spinal Anesthesia: A Study of 3492 Patients. **Journal of Laparoendoscopic & Advanced Surgical Techniques** 2009; 19:323–327.

Steiner LA. Postoperative delirium. Part 1: pathophysiology and risk factors. **European Journal of Anaesthesiology** 2011a; 28:628–636.

Steiner LA. Postoperative delirium. Part 2: detection, prevention and treatment. **European Journal of Anaesthesiology** 2011b; 28:723–732.

Talab SS, Elmi A, Sarma J, Barrisford GW, Tabatabaei S. Safety and Effectiveness of SAF-R, a Novel Patient Positioning Device for Robot-Assisted Pelvic Surgery in Trendelenburg Position. **Journal of endourology / Endourological Society** 2016; 30:286–92.

Veronesi U, Stafyla V. Grand challenges in surgical oncology. 2012; 2:1–3. Wang M. Optimal Depth for Nasopharyngeal Temperature Probe Positioning.

**Anesthesia and Analgesia** 2016;
Whitlock E, Vannucci A, Avidan M. Postoperative delirium. **Minerva**

**Anaesthesiology** 2011; 77:448–456.
Wong CL, Holroyd-Leduc J, Simel DL, Straus SE. Does this patient have

delirium?value of bedside instruments. **JAMA** 2010; 304:779.

Wongyingsinn M, Baldini G, Stein B, Charlebois P, Liberman S, Carli F. Spinal analgesia for laparoscopic colonic resection using an enhanced recovery after surgery programme: Better analgesia, but no benefits on postoperative recovery: A randomized controlled trial. **British Journal of Anaesthesia** 2012; 108:850–856.

**SUPPLEMENT 1 – Confusion Assement Method - CAM**

**Confusion Assement Method - CAM**

**1. Acute onset**

There is evidence of acute change in the underlying mental state of the patient? (YES NO)

**2. Attention disorder**

Did the patient have difficulty focusing his attention, for example, easily distracted or had difficulty keeping up with what was being said? (YES) (NO)

**3. Disorganized Thinking**

Was the patient's thinking disorganized or inconsistent, with scattered or irrelevant conversion, unclear or illogical flow of ideas, or unpredictable change of subject? (YES) (NO)

**4. Change in level of consciousness**

Does the patient have altered level of consciousness such as lethargy, numb, comatose? (YES) (NO)

**Criteria 1 and 2 plus 3 or 4 must be present.**

Adapted from: (INOUYE SK, VAN DICK CH, ALESSI CA 1990)

**SUPPLEMENT 2 - Richmond Agitation Sedation Scale - RASS Richmond Agitation Sedation Scale - RASS**

| **Score** | **Term** | **Description** |
| --- | --- | --- |
| + 4 | Combative | Overtly combative or violent; immediate danger to staff |
| + 3 | Very agitation | Pulls on or removes tube(s) or catheter(s) or has aggressive behavior toward staff |
| + 2 | Agitated | Frequent nonpurposeful movement or patient–ventilator dyssynchrony |
| + 1 | Restless | Anxious or apprehensive but movements not aggressive or vigorous |
| 0 | Alert and calm |  |
| - 1 | Drowsy | Not fully alert, but has sustained (more than 10 seconds) awakening, with eye contact, to voice |
| - 2 | Light sedation | Briefly (less than 10 seconds) awakens with eye contact to voice |
| - 3 | Moderate sedation | Any movement (but no eye contact) to voice |
| - 4 | Deep sedation | No response to voice, but any movement to physical stimulation |
| - 5 | Unarousable | No response to voice or physical stimulation |

Source: Adapted from: (SESSLER et al. 2002).
